# Selective use of distant stone resources by the earliest Oldowan toolmakers

**DOI:** 10.1126/sciadv.adu5838

**Published:** 2025-08-15

**Authors:** Emma M. Finestone, Thomas W. Plummer, Peter W. Ditchfield, Jonathan S. Reeves, David R. Braun, Simion K. Bartilol, Nelson Kiprono Rotich, Laura C. Bishop, James S. Oliver, Rahab N. Kinyanjui, Michael D. Petraglia, Paul S. Breeze, Cristina Lemorini, Isabella Caricola, Paul Owich Obondo, Richard Potts

**Affiliations:** ^1^ Cleveland Museum of Natural History, Cleveland, OH, USA.; ^2^ Department of Anthropology, Queens College, Flushing, NY, USA.; ^3^ CUNY Graduate Center, New York, NY, USA.; ^4^ Human Origins Program, National Museum of Natural History, Smithsonian Institution, Washington DC, USA.; ^5^ New York Consortium in Evolutionary Primatology, New York, NY, USA.; ^6^ School of Archaeology, University of Oxford, Oxford, UK.; ^7^ Interdisciplinary Center for Archaeology and the Evolution of Human Behavior, Campus Gambelas, Universidade do Algarve Faro, Portugal.; ^8^ Technological Primates Research Group, Max Planck Institute for Evolutionary Anthropology, Leipzig, Germany.; ^9^ George Washington University, Washington DC, USA.; ^10^ Institute of Nuclear Science and Technology, University of Nairobi, Nairobi, Kenya.; ^11^ Institute of Nuclear Chemistry and Technology, Warsaw, Poland.; ^12^ University of Nottingham Ningbo China, Ningbo, People’s Republic of China.; ^13^ Anthropology Section, Illinois State Museum, Springfield, IL, USA.; ^14^ Department of Earth Sciences, National Museums of Kenya, Nairobi, Kenya.; ^15^ Australian Research Centre for Human Evolution, Griffith University, Brisbane, Australia.; ^16^ School of Social Science, University of Queensland, Brisbane, Australia.; ^17^ Department of Geography, King’s College London, London, UK.; ^18^ LTFAPA Laboratory, Department of Classics, Sapienza University of Rome, Rome, Italy.; ^19^ Zinman Institute of Archaeology, Haifa University, Haifa, Israel.; ^20^ Department of Anthropology, University of Nairobi, Nairobi, Kenya.

## Abstract

The adaptive shift that favored stone tool–assisted behavior in hominins began by 3.3 million years ago. However, evidence from early archaeological sites indicates relatively short-distance stone transport dynamics similar to behaviors observed in nonhuman primates. Here we report selective raw material transport over longer distances than expected at least 2.6 million years ago. Hominins at Nyayanga, Kenya, manufactured Oldowan tools primarily from diverse nonlocal stones, pushing back the date for expanded raw material transport by over half a million years. Nonlocal cobbles were transported up to 13 kilometers for on-site reduction, resulting in assemblage patterns inconsistent with accumulations formed by repeated short-distance transport events. These findings demonstrate that early toolmakers moved stones over substantial distances, possibly in anticipation of food processing needs, representing the earliest archaeologically visible signal for the incorporation of lithic technology into landscape-scale foraging repertoires.

## Teaser

Toolmakers selectively transported a diversity of stone materials at least 2.6 million years ago.

## INTRODUCTION

Humans and their ancestors are the only mammals known to procure lithic resources over long distances. As early as 2 million years ago (Ma), hominins routinely carried stone tools across the landscape to process foods in areas lacking hard and durable raw materials nearby (*1*). This emerging system of lithic transport signified a major breakthrough in the human lineage in terms of mental mapping capabilities, land-use strategy, and the behavioral flexibility to access a range of previously unexploited resources (*2*, *3*). Although stones have no inherent caloric value, their acquisition became increasingly central to hominin foraging for plant and animal tissue through time.

A variety of animals exhibit long-distance spatial resource use (*4*, *5*) and occasionally modify their behavior in anticipation of future circumstances (*6*–*8*). Human behavior, however, is unusual if not unique in the degree of forward planning (*9*, *10*) [i.e., the ability to make decisions about future events that will unfold at other locations (*6*)] and the delay incurred between foraging efforts and food consumption (*11*). The preferential transport of stones to locations on the landscape for food processing is perhaps the first archaeologically visible indicator of behavioral strategies that imply elements of planning and delayed payoff (*9*, *12*, *13*). Recognizing early traces of lithic resource movement is important for understanding when and how hominins began incorporating stone material into their foraging routine and mental maps. The reorganization of food resources and stone tools across landscapes signaled a shift in resource exploitation that broadened foraging opportunities and facilitated food sharing on a scale unknown in other primates (*14*).

**Fig. 1.**
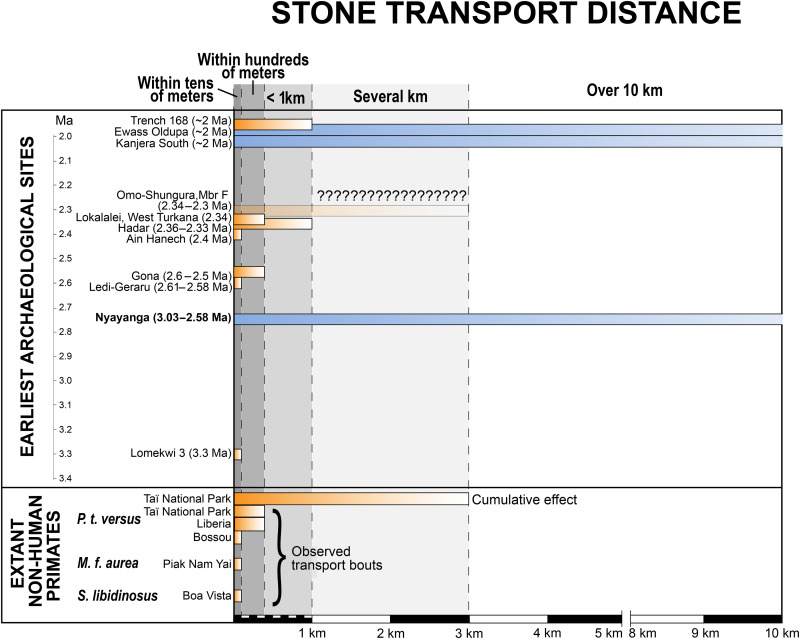
Evidence of stone transport in the archaeological record. Published estimates of raw material distance at the earliest archaeological occurrences (2 million years and older) from Trench 168, Olduvai Gorge (*81*); Ewass Oldupa, Tanzania (*46*); Kanjera South, Kenya (*1*); Omo Shungura Formation; Member F, Ethiopia (*44*, *82*) (? = raw material sources not identified); Lokalalei, West Turkana, Kenya (*15*); Hadar, Ethiopia (*16*); Ain Hanech, Algeria (*42*); Zarqa Valley, Jordan (*83*); Gona, Ethiopia (*23*); Nyayanga, Kenya (this study); and Lomekwi 3, Kenya (*40*). These are compared to nonhuman primate stone transport distances observed during transport bouts at Taï National Park, Côte d’Ivoire (*33*), a released population on an island in Liberia (*84*); Bossou, Guinea (*28*); Piak Nam Yai island, Thailand (*85*); and Boa Vista, Brazil (*30*) and cumulative effects at Taï (*26*). Occurrences with transport distances more than 10 km are shaded blue and less than 10 km are shaded orange.

The earliest traces of hominin stone tool behavior appear to lack evidence of sophisticated resource transport. Before 2 Ma, hominins were selective in the raw materials they used to make tools. However, early archaeological accumulations were routinely found near naturally occurring sources of stone (*15*–*19*), and transport distances were generally a few meters to a few hundred meters (Fig. 1) (*15*, *16*, *20*–*25*). These acquisition and transport behaviors are within the range of extant nonhuman primate activities (Fig. 1). Modern primates are sensitive to mechanical properties when selecting stones for percussion (*26*, *27*–*31*) and are known to selectively use and transport stone tools over equivalent distances to early hominin toolmakers (*26*–*30*, *32*, *33*). For instance, chimpanzees can move stone tools over 2 km through cumulative short distance transport bouts during artifact use and reuse (*26*).

The accumulation of unplanned, undirected, short-distance tool transport events results in a distance-decay relationship (*26*, *34*), which is defined as a positive correlation between the level of raw material utilization in a tool assemblage and the distance to that raw material source. This pattern occurs under neutral conditions in the absence of factors such as habitat preferences and advanced planning (*35*–*37*). At some of the earliest Oldowan sites, the landscape scale distribution of lithics can be explained by repeated short-distance transport events and distance decay effects (*35*, *38*). However, the use-life duration of core and flake technology as well as the environmental context of Early Stone Age sites differ from the conditions seen in nonhuman primate percussive tool-use, limiting their applicability for modeling Oldowan tool transport (*39*).

**Fig. 2.**
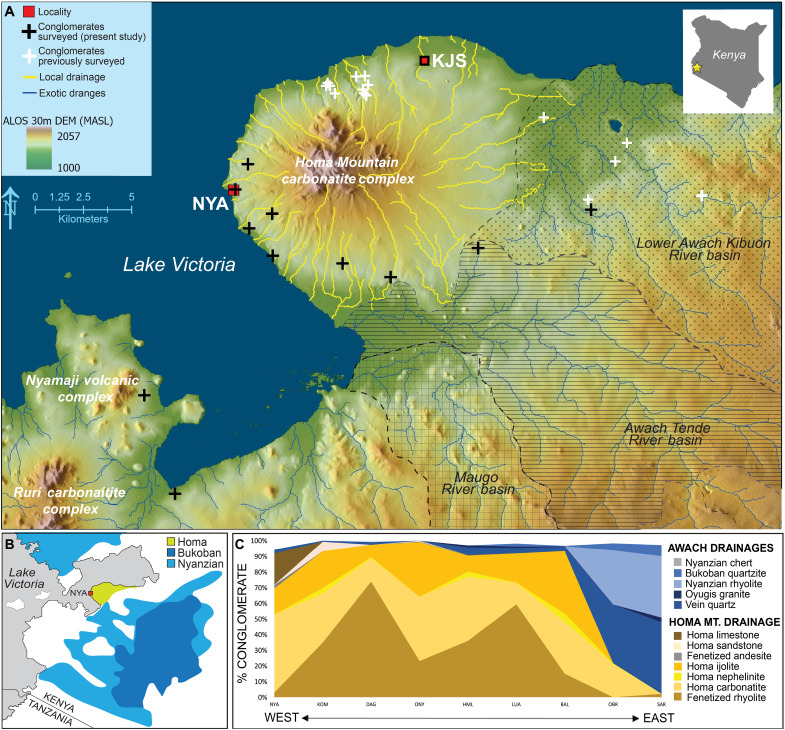
Drainage systems around the Homa Peninsula, Kenya. (**A**) Map of the Homa Peninsula with a model of the extent of the Homa Mountain and eastern drainages (Maugo, Awach Tende, and Lower Awach Kibuon rivers) (*86*). Nyayanga (NYA), Kanjera South (KJS) and the locations of conglomerates sampled in this study and previously studied by Braun *et al.* (*1*) are marked. (**B**) Schematic of the Homa Mountain (yellow), Bukoban (dark blue), and Nyanzian (light blue) rock supergroups (*87*). Lake Victoria (shaded gray) and the Nyayanga locality (NYA) are labeled. (**C**) The rock types found in the nine conglomerates sampled in this study from the Awach and Homa Mountain (Homa Mt.) drainage networks are shown. The percentage of each rock type is presented from west to east, corresponding to their proximity to the Nyayanga locality. NYA, Nyayanga; HML, Homa Lime; BAL, Bala; DAG, Dago; SAR, Sare Abururu; LUA, Luanda West; OBR, Oboro; KOM, Komullo; ONY, Onyango; KIS, Kisaka; KAN, Kananga.

Hominin resource transport appears to have diverged from patterns typical of extant nonhuman primate tool procurement by 2 Ma (Fig. 1). The earliest evidence of long-distance raw material transport (defined here as >3 km, i.e., beyond what is seen in nonhuman primates from cumulative transport bouts) is documented at the Oldowan locality Kanjera South, on the Homa Peninsula, Kenya (*1*) (Fig. 2) and, more recently, at Ewass Oldupai, Tanzania (*40*). Research from Kanjera South demonstrated that hominins traveled over 10 km to access durable, hard, and brittle (i.e., easily fractured by intentional lithic knapping) raw materials including quartzite, rhyolite, quartz, chert and granite, as the locally available lithologies on the Homa Peninsula were of comparatively low durability (*1*, *41*–*43*).

While it is possible that early toolmakers sometimes gathered resources from widely dispersed locations across the landscape before 2 Ma, there is limited archaeological evidence to support this. The small number of archaeological localities older than 2 Ma (*21*, *44*–*49*) likely fails to capture the full range of variation in habitats, toolmaker niches, and stone resource distribution in the early Oldowan, making it difficult to interpret hominin resource movement at the dawn of technology. Data from early artifact assemblages situated in different ecological conditions and farther from stone sources are needed to assess whether there is greater behavioral variation in early stone resource transport than is currently recognized.

Nyayanga, Kenya (3.040 to 2.581 Ma) (*50*) located 11.4 km to the west of Kanjera South, preserves an early Oldowan assemblage associated with *Paranthropus* remains and multiple hippopotamid butchery sites (*50*). Nyayanga provides an opportunity to examine stone transport dynamics at the emergence of the Oldowan within the same landscape distribution of stone lithologies investigated at Kanjera South, where high-quality stone materials are not locally abundant. If patterns of stone resource movement at Nyayanga are similar to the simple acquisition strategies and short transport distances observed at other early localities (Fig. 1), then toolmakers likely did not transport stone over long distances during the early Oldowan, even in areas lacking hard raw materials locally. Alternatively, if the stone tools deposited by hominins at Nyayanga were moved over longer distances and do not reflect distance-decay patterns, this would extend the timing of long-distance raw material transport back at least 600,000 years and suggest that long-distance resource procurement was within the scope of early Oldowan behavioral variability.

## RESULTS

### The Nyayanga artifact assemblage

Artifacts included in this study (*n* = 401) were collected from excavations 3 (*n* = 115), 5 (*n* = 14), and from surface survey (*n* = 272) targeting the upper half of bed NY-1. Most of the tools from excavations 3 and 5 were recovered in fine-grained sedimentary layers primarily composed of silts, and, less frequently, sandy silts and fine sands, indicating rapid burial by fluvial sediments (*50*). Artifacts exhibit excellent surface preservation with relatively fresh and well-preserved edges. The presence of cortical flakes (*n* = 11) and hammerstones with battering damage (*n* = 16) suggests that on-site flake production via hard hammer percussion took place at the Nyayanga locality.

The Nyayanga assemblage consists of detached pieces (*n* = 294, 73.3%), flaked pieces (*n* = 88, 21.9%), and pounded pieces (*n* = 16, 4.0%). Several manuports are also present (*n* = 3). The cores from Nyayanga are relatively large for the Oldowan Industry, with an average weight of 374.8 g (SD = 364.7) and an average maximum dimension of 77.7 g (SD = 24.1). An unusually large number of artifacts—both pounded pieces and flaked pieces—preserve evidence of percussive activity (*n* = 33, 8.2%). In other Oldowan assemblages, cores typically account for 1 to 16% of the assemblage, and artifacts with percussion damage generally represent only 1 to 3% (*50*).

### Raw materials available around the Homa Peninsula, Kenya

**Fig. 3.**
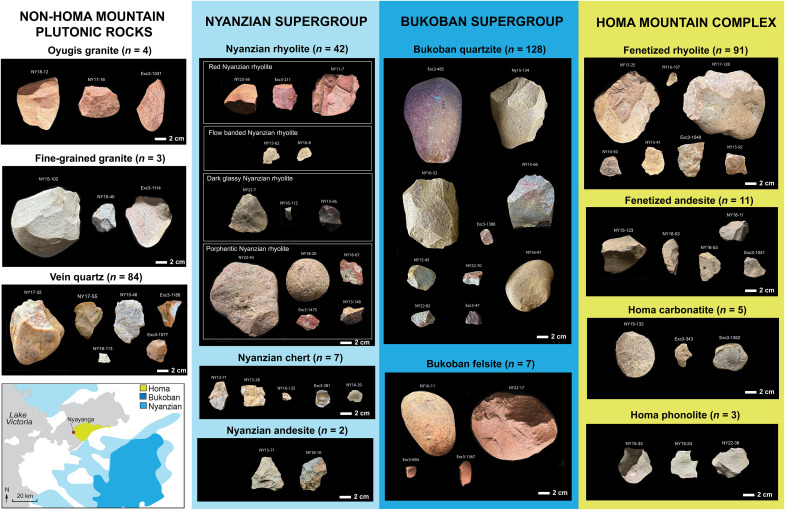
Select Nyayanga artifacts organized by raw material and geological supergroup. Artifacts are pictured from common raw materials that derive from the Nyanzian (light blue) and Bukoban (dark blue) rock supergroups, the Homa Mountain complex (yellow), and non–Homa Mountain plutonic rocks (white). A map of the Homa Mountain (yellow), Bukoban (dark blue), and Nyanzian (light blue) rock supergroups is shown on the lower left (*87*).

Survey of outcropping conglomerates and previous geological mapping and geochemical analyses (*51*–*53*) support a division through the duration of the Plio-Pleistocene between the local lithologies available on the Homa Peninsula and the nonlocal clasts that originate from Precambrian highlands to the east and are not readily available on the peninsula through the duration of the Plio-Pleistocene. The rock types commonly used in Nyayanga tool manufacture derive primarily from three geological systems in the region: the Homa Mountain carbonatite center, the Nyanzian Supergroup, and the Bukoban Supergroup (Figs. 2 and 3). The most ancient of these, the Nyanzian, includes rhyolites, dacites, andesites, and cherts. The Nyanzian Supergroup of lavas was widely emplaced over the region and formed a peneplain before the doming of the Homa Mountain complex from carbonatite volcanism in the Miocene (*51*). Around Homa Mountain, these Nyanzian clasts underwent intense metasomatism, producing fenetized Nyanzian rocks that are softer and less durable than their unfenetized counterparts (*49*). Fenetized clasts, along with Homa Mountain ijolite, carbonatite, and limestone, are common in the Homa Mountain complex. Last, the Bukoban Supergroup is composed of lavas and metamorphic sediments that were emplaced hundreds of millions of years ago across highlands dozens of kilometers southeast of the Homa Peninsula. Bukoban rocks include basalts, felsites, and banded fine-grained quartzites that range from gray to dark red. Bukoban rocks are not found in fenetized form anywhere on the peninsula, indicating that these were not present during the volcanic activity of the Homa Mountain and therefore did not undergo the intense metasomatic alteration (fenetization) observed in Nyanzian rocks.

River systems transport clasts from the Homa Mountain, Nyanzian, and Bukoban Supergroups throughout the region. The system local to the peninsula, the Homa Mountain radial drainage network, has been in place since the Miocene and deposits clasts predominately derived from the mountain’s carbonatite complex across the peninsula. In contrast, river systems to the east carry nonlocal lithologies toward the peninsula from the Nyanzian and Bukoban eastern highlands (*1*). The eastern drainages follow regional faults that existed since the early Pliocene based on the estimated age of emplaced lavas (*51*, *54*–*56*). Two river systems, in particular—the Awach Tende and Lower Awach Kibuon—transport river cobbles from durable materials such as Bukoban quartzite, Nyanzian rhyolite, vein quartz, and Oyugis granite from eastern primary outcrops toward, but not onto the Homa Peninsula. The Awach rivers end at the margin of the peninsula because their flow is directed around the topographic high of Homa Mountain and overpowered by the energy of the Homa Mountain radial drainage network, which was even greater during the Pliocene (*1*, *51*, *52*). This created a natural division in raw materials between the local Homa Mountain carbonatite complex lithologies in the peninsular radial drainage system and the nonlocal materials available in the Awach drainages. This division persisted for millions of years and is reflected in conglomerates formed across a range of geological epochs. Because the Nyanzian and Bukoban Supergroups are Precambrian and have been unroofed and exposed for tens of millions of years, their composition in conglomerates has changed very little from the middle Pliocene to the present (Fig. 1 and table S1) (*1*).

Previous work has focused on conglomerates from the northern half of the peninsula and the Lower Awach Kibuon river (Fig. 2). Here, 11 conglomerates were analyzed because of their accessibility to Nyayanga hominins and because they are representative of drainages on the previously unsampled southern half of the Homa Peninsula, the Awach Tende river, and the flanks of volcanic complexes west of the peninsula (Fig. 2, fig. S1, and Table 1). Four to eight of these conglomerates were coeval to beds in the Nyayanga sequence. The others are Pleistocene age, and one is modern, but all reflect the regional availability of lithologies for Nyayanga hominins.

**Table 1.** Conglomerates surveyed in this study. Their drainage system (Homa Mt., Homa Mountain radial drainage), straight line distance (kilometers) to the Nyayanga excavations, geological formation, and previous descriptions are noted.

Locality	Drainage system	Distance from Nyayanga	Geological formation	Previous geological description
**Nyayanga**	Homa Mt.	0.09 km	NY-1 Bed, Late Pliocene	Plummer *et al.* (*50*)
**Komullo**	Homa Mt.	1.52 km	Plio-Pleistocene	-
**Dago**	Homa Mt.	2.16 km	NY-1 Bed, Late Pliocene	Le Bas (*51*)
**Onyango**	Homa Mt.	2.35 km	Unknown	-
**Homa Lime**	Homa Mt.	3.95 km	Homa Fm, Late Pliocene	Saggerson, (*52*)
**Luanda**	Homa Mt.	6.82 km	Homa Fm, Late Pliocene	Tryon *et al.* (*63*); Blegen *et al.* (*64*)
**Bala**	Homa Mt.	9.30 km	Homa Fm (?), Late Pliocene	Pickford, (*88*)
**Kisaka**	Wasaki Peninsula	11.58 km*	Pleistocene	-
**Oboro**	Awach River (Awach Tende)	13.06 km	Modern	-
**Kananga**	Wasaki Peninsula	15.98 km*	Plio-Pleistocene	-
**Sare-Abururu**	Awach River (Lower Awach Kibuon)	18.61 km	Pleistocene (Orio Tuff ~1.7 Ma)	Le Bas, (*51*); Finestone *et al.* (*65*)

*Straight line distance calculated across the Homa Bay.

The composition of these conglomerates is consistent with those previously described for drainages to the north and east (*1*, *49*). Rock types that originate from the Homa Mountain carbonatite complex were abundant in paleo-conglomerates near Nyayanga, including high frequencies of fenetized Nyanzian rhyolite, carbonatite, ijolite, and sandstone. These clasts were predominantly subangular or angular blocks, reflecting their short duration of fluvial transport from Homa Mountain primary sources before their deposition in conglomerates (Table 2). In contrast, the lithologies that are known to originate east of the Homa Peninsula (e.g., Nyanzian chert, Bukoban quartzite, Nyanzian rhyolite, Oyugis granite, and quartz) were absent or very rare and of small size in conglomerates on the peninsula but were common and of greater size in conglomerates further east within the Awach drainage networks (Figs. 1 and 2). Clasts from the Awach conglomerates were predominately rounded or subrounded river cobbles, demonstrating a longer fluvial transport history than Homa Mountain rock types (Table 2).

**Table 2.** Angularity of conglomerates surveyed in this study. The percent of angular, subangular, subrounded and rounded cobbles in each conglomerate are reported. The most common is shown in bold for each conglomerate.

Locality of conglomerate	Drainage system	Angular	Subangular	Subrounded	Rounded	Total
**Homa Lime**	Homa Mt.	**40.55%**	37.28%	17.63%	4.53%	397
**Onyango**	Homa Mt.	18.04%	**60.13%**	19.94%	1.90%	316
**Dago**	Homa Mt.	29.09%	**54.14%**	14.14%	2.63%	495
**Luanda**	Homa Mt.	30.62%	**56.46%**	12.92%	0.00%	209
**Bala**	Homa Mt.	20.88%	**52.38%**	23.44%	3.30%	273
**Komullo**	Homa Mt.	38.93%	**48.32%**	12.08%	0.67%	149
**Kananga**	Wasaki Peninsula	33.20%	**60.00%**	6.80%	0.00%	250
**Kisaka**	Wasaki Peninsula	7.99%	**78.99%**	13.02%	0.00%	338
**Oboro**	Awach Rivers	8.21%	33.68%	**45.47%**	12.63%	475
**Sare**	Awach Rivers	2.86%	33.33%	**60.00%**	0.00%	499

### Assemblage raw material selectivity

When Nyayanga artifact (*n* = 401) raw materials are compared to the frequency distribution of rock types, a pattern of high selectivity in hominin toolmaking emerges (Table 3). Most of the tools (>70%) were manufactured from nonlocal materials that originated east of the peninsula and were available primarily in the eastern drainage system. Artifacts were manufactured from a diversity of raw materials, with the most frequent being Bukoban quartzite (*n* = 130; 32.4% of assemblage), fenetized Nyanzian rhyolite (*n* = 94; 23.4%), quartz (*n* = 84; 20.9%), and Nyanzian rhyolite (*n* = 43; 10.7%). Nyanzian chert, Oyugis granite, Bukoban felsite, fenetized andesite, and Homa Mountain carbonatite were also present in the assemblage at low frequencies (between 1 and 2% each).

**Table 3.** The difference between expected counts of raw materials versus observed counts in the Nyayanga artifact assemblage. Expected raw material frequencies are derived from the average percent of each lithology in conglomerates within that drainage system. Chi-square tests indicate significant differences between the observed.

	*Raw material*	*Observed*	*Expected*
Homa Mountain drainage*	Fenetized rhyolite	94	31
	Homa carbonatite	5	33
	Homa nephelinite	0	2
	Homa ijolite	0	22
	Fenetized andesite	11	0
	Homa sandstone	0	1
	Homa limestone	0	3
Awach drainages†	Vein quartz	84	112
	Oyugis granite	7	4
	Nyanzian rhyolite	43	99
	Bukoban quartzite	130	15
	Nyanzian chert	7	1

*Chi-square test of observed versus expected Homa Mountain raw materials in the Nyayanga assemblage: χ^2^ (6) = 90.5, *P* < 0,001.

†Chi-square test of observed versus expected Awach raw materials in the Nyayanga assemblage: χ^2^ (4) = 120.2, *P* < 0,001.

**Fig. 4.**
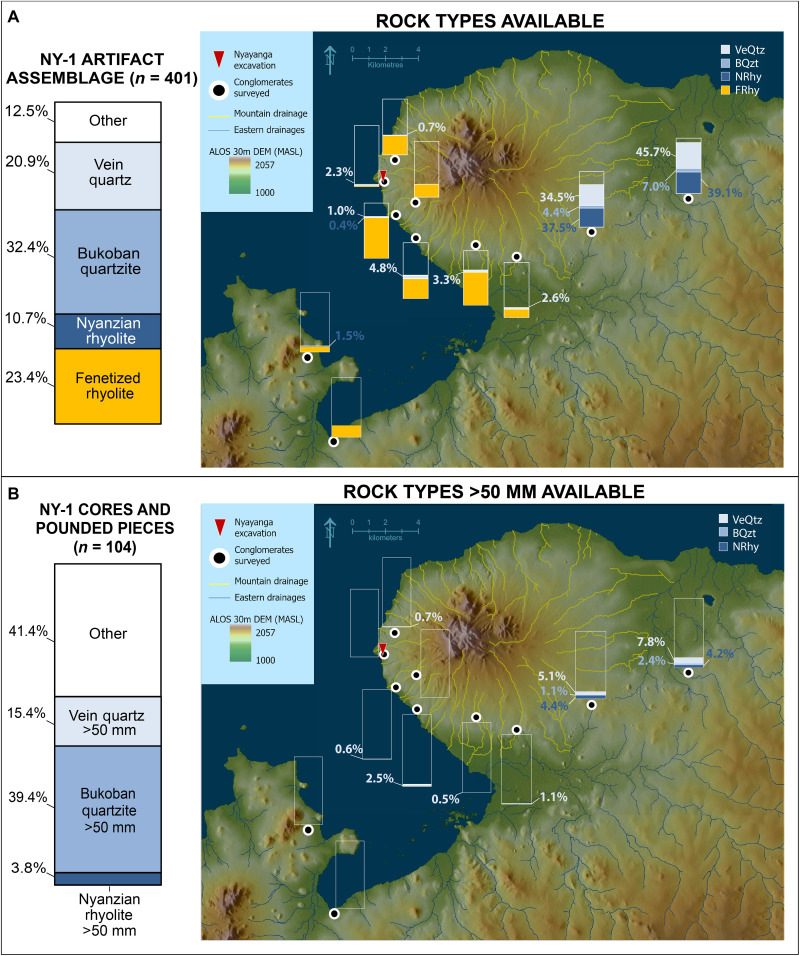
The availability of stones used by Nyayanga hominins to make tools. (**A**) The total percentage of Vein quartz (VeQtz), Bukoban quartzite (BQzt), Nyanzian rhyolite (NRhy), and Fenetized rhyolite (FRhy) lithics in the Nyayanga assemblage compared to the percentage of naturally occurring cobbles available in the 11 conglomerates sampled. (**B**) The percentage of Nyayanga cores and hammerstones made from Nyanzian rhyolite, Bukoban quartzite, and Vein quartz measuring over 50 mm in maximum dimension recovered in Bed NY-1 compared to the percentage of Nyanzian rhyolite, Bukoban quartzite, and Vein quartz rock types over 50 mm present in sampled conglomerates. “Other” includes all cores under 50 mm in maximum dimension or cores manufactured from materials other than Nyanzian rhyolite, Bukoban quartzite, and Vein quartz.

Nyayanga hominins were selective, using relatively large clasts from the available cobbles. Most cores in the assemblage (86.4%, *n* = 76) exceed 50 mm in maximum dimension (Table 4). Quartz cobbles of this size are rare in Homa Peninsula conglomerates (Fig. 4), while Bukoban quartzite and Nyanzian rhyolite cobbles of this size are entirely absent. Among the selected materials, quartzite tools were manufactured from the largest clasts. Quartzite cores (*n* = 32) are the largest of any raw material in the assemblage (mean = 85.2 mm, SD = 23.3). Quartzite tools (*n* = 130) from Nyayanga are also significantly greater in mass (mean = 210.8 g, SD = 328.3) when compared to the rest of the artifact assemblage (*n* = 271; mean = 89.1 g, SD = 209.0; two-sample *t* test: *t*(399) = −4.50, *P* < 0.001). All quartzite cobbles brought to Nyayanga as manuports (*n* = 2) and pounded pieces (n = 13) exceeded 50 mm in maximum dimension (mean = 104.1 mm, SD 15.7).

**Table 4.** Weights and maximum dimensions of artifacts in the Nyayanga assemblage. Count, percent, maximum dimension, and weight are reported for each artifact type and raw materials. Abbreviations: FP, flaked piece; DP, detached piece; PP, pounded piece.

Artifact type	Raw material origin	Raw material	Count	Percent	Maximum dimension (mm)	Maximum dimension (SD)	Weight (g)	Weight (SD)
***DP***	Nonlocal	Bukoban quartzite	83	20.7%	38.5	14.7	20.7	35.0
		Vein quartz	64	16.0%	33.1	13.6	14.7	19.4
		Nyanzian rhyolite	38	9.5%	37.2	12.7	15.5	19.0
		Nyanzian chert	5	1.2%	35.4	14.2	10.1	9.7
		Bukoban red felsite	4	1.0%	33.4	10.7	7.4	4.8
		Oyugis granite	1	0.2%	23.5	-	3.4	-
	Local	Fenetized rhyolite	74	18.5%	44.4	15.6	28.6	39.6
		Fenetized andesite	9	2.2%	41.5	13.8	15.9	11.0
		Homa carbonatite	4	1.0%	57.8	17.3	77.2	98.0
		Assemblage total	294	73.3%	39.4	15.2	21.2	32.5
***FP***	Nonlocal	Bukoban quartzite	32	8.0%	85.2	23.3	496.1	366.8
		Vein quartz	20	5.0%	69.5	20.0	246.3	240.0
		Nyanzian rhyolite	3	0.7%	84.9	35.6	407.8	368.0
		Nyanzian chert	2	0.5%	39.4	12.3	19.8	13.9
		Bukoban red felsite	1	0.2%	139.1	-	1595.0	-
		Oyugis granite	3	0.7%	88.3	20.2	333.5	267.9
	Local	Fenetized rhyolite	19	4.7%	71.7	22.2	278.8	312.3
		Fenetized andesite	2	0.5%	62.3	15.0	136.8	96.7
		Homa carbonatite	1	0.2%	82.8	-	186.4	-
		Assemblage total	88	21.9%	77.8	24.1	374.8	364.7
***PP***	Nonlocal	Bukoban quartzite	13	3.2%	102.6	16.2	614.3	276.0
		Vein quartz	0	0.0%	-	-	-	-
		Nyanzian rhyolite	1	0.2%	73.0	-	409.0	-
		Nyanzian chert	0	0.0%	-	-	-	-
		Bukoban red felsite	2	0.5%	116.4	1.6	826.5	147.8
		Oyugis granite	0	0.0%	-	-	-	-
	Local	Fenetized rhyolite	0	0.0%	-	-	-	-
		Fenetized andesite	0	0.0%	-	-	-	-
		Homa carbonatite	0	0.0%	-	-	-	-
		Assemblage total	16	4.0%	102.5	17.1	628.0	266.5
***Manuport***	Nonlocal	Bukoban quartzite	2	0.5%	114.3	6.6	916.5	13.4
		Vein quartz	0	0.0%	-	-	-	-
		Nyanzian rhyolite	1	0.2%	83.7	-	443.8	-
		Nyanzian chert	0	0.0%	-	-	-	-
		Bukoban red felsite	0	0.0%	-	-	-	-
		Oyugis granite	0	0.0%	-	-	-	-
	Local	Fenetized rhyolite	0	0.0%	-	-	-	-
		Fenetized andesite	0	0.0%	-	-	-	-
		Homa carbonatite	0	0.0%	-	-	-	-
		Assemblage total	3	0.7%	104.1	18.3	758.9	273.1

### Sources accessed by hominins

**Fig. 5.**
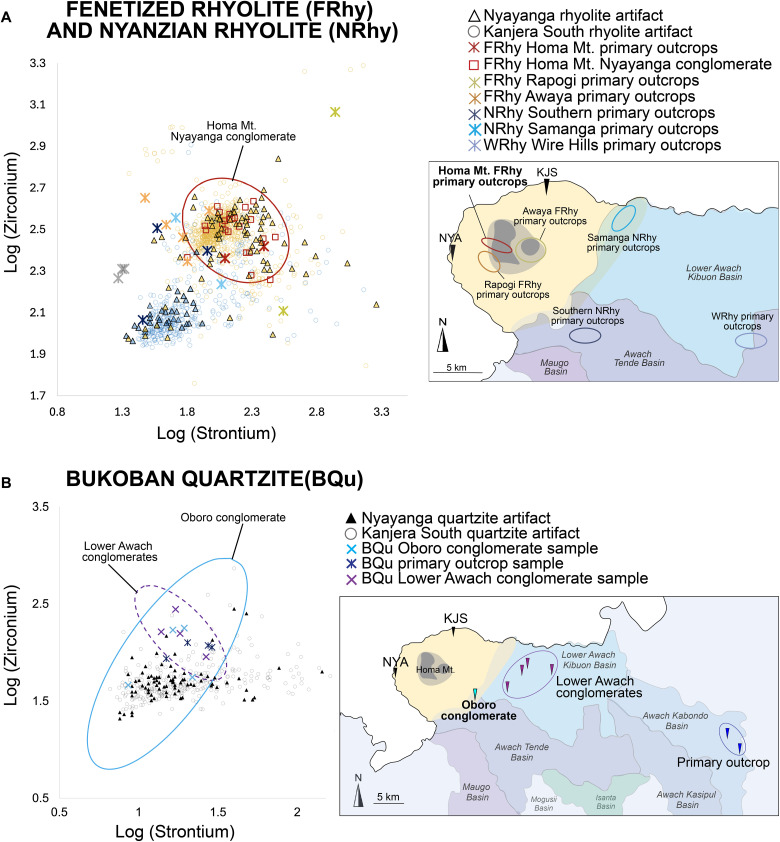
Rhyolite and quartzite artifacts from Nyayanga and Kanjera South linked to sources. (**A**) The ratio of log_10_ (strontium) (*x* axis) and log_10_ (zirconium) (*y* axis) concentrations in parts per million for all artifacts attributed to rhyolite from the Nyayanga (triangle) and Kanjera South (open circle) assemblages. Fenetized Nyanzian artifacts from Nyayanga (*n* = 78) and Kanjera South (*n* = 755) are shaded yellow. Unfenetized Nyanzian rhyolite artifacts from Nyayanga (*n* = 33) and Kanjera South (*n* = 356) are shaded blue. Strontium and zirconium geochemistry of Homa Mt. (red), Rapogi (yellow), and Awaya Hills (orange) primary outcrops of fenetized rhyolite (FRhy) and Wire Hills (gray), Southern (dark blue), and Samanga (light blue) primary outcrops of Nyanzian rhyolite (NRhy) are also plotted. A 95% confidence ellipse for Homa Mountain (Homa Mt.) secondary sources of fenetized rhyolite from the Nyayanga local conglomerate (red boxed) is overlaid. A map of these sources of rhyolite around the Homa Peninsula is also displayed. (**B**) The ratio of log_10_ (strontium) (*x* axis) and log_10_ (zirconium) (*y* axis) concentrations in parts per million for all artifacts attributed to quartzite from the Nyayanga (triangle, *n* = 82) and Kanjera South (open circle, *n* = 290) assemblages. Strontium and zirconium geochemistry of Bukoban quartzite (BQu) primary sources (dark blue), Lower Awach Kiboun secondary conglomerates identified by Braun *et al.* (*1*) (purple), and Oboro conglomerate quartzite (light blue) are plotted. A 95% confidence ellipse for the Lower Awach Kiboun and Oboro quartzite conglomerates are overlain.

Trace element geochemistry of a subset of fenetized rhyolite (*n* = 81), Nyanzian rhyolite (*n* = 35), and quartzite (*n* = 86) artifacts link them to sources in the vicinity of the Homa Peninsula (figs. S4 to S6). Strontium and zirconium are the elements with the highest variability and are therefore the most useful for distinguishing between raw material sources (*8*, *53*). The strontium and zirconium composition of Nyayanga quartzite artifacts are most similar to the geochemical signatures of quartzite clasts available in the Oboro conglomerate of the Awach Tende river basin (Fig. 5), suggesting that Nyayanga hominins likely accessed sources of quartzite in the vicinity of Oboro.

Quartzite cobbles available in the Oboro drainage of the Awach Tende river network exhibit greater variability in zirconium parts per million (ppm) and lower concentrations of rubidium and yttrium than secondary sources of quartzite in the Lower Awach Kibuon basin (Fig. 5 and fig. S4). The Nyayanga quartzite assemblage have similar geochemistry; 70 of the 82 quartzite artifacts with measurable strontium and zirconium values overlap with the 95% confidence ellipse of the Oboro conglomerate sample. This contrasts with secondary outcrops of Bukoban quartzite previously identified by Braun *et al.* (*1*) in the Lower Awach Kibuon basin. Only six Nyayanga artifacts fall within the 95% confidence ellipse for the Lower Awach Kibuon quartzite sample, and most of these also fall within with the Oboro confidence ellipse (Fig. 3). When comparing trace element geochemistry across all four elements analyzed (Rb, Sr, Y, and Zr), the Nyayanga artifacts show greater geochemical similarity to the Oboro conglomerate than to secondary outcrops in the Lower Awach Kibuon basin (fig. S4).

Most fenetized Nyanzian rhyolite artifacts appear to derive from primary outcrops at the center of the Homa Mountain (Fig. 5). The Nyayanga local conglomerate provided a secondary source of these cobbles, and their geochemistry closely reflects the artifact sample, suggesting that Nyayanga hominins used the nearest sources of fenetized rock (Fig. 5 and fig. S5). A handful of fenetized Nyanzian rhyolite artifacts show lower elemental concentrations, likely due to intense fenetization. In such cases, secondary minerals can form along joint surfaces and penetrate the rock, gradually replacing it and altering the trace element geochemistry.

The geochemistry of unfenetized Nyanzian rhyolite stone tools is more similar to southern sources of rhyolite compared with northern sources (Fig. 5 and fig. S6). However, the degree of variability in the appearance (Fig. 3) and geochemistry (Fig. 5) of Nyanzian rhyolite artifacts suggests that these rocks may have been obtained from multiple sources.

Nyayanga artifacts made from other raw materials (i.e., quartz, granite, Nyanzian chert, Homa carbonatite, fenetized andesite, and Bukoban felsite) were also analyzed using energy-dispersive x-ray fluorescence (ED-XRF) (*n* = 106) and exhibited variable trace element geochemistry (fig. S7). However, we could not link these materials to specific sources due to small sample sizes and the challenge of distinguishing between quartz sources geochemically.

### Stone transport distance

Transport distances were inferred by examining the presence and absence of rock types in the sampled conglomerates. Although Nyayanga is 600 thousand years (ka) older, the tool transport distances at the site are comparable to Kanjera South (*1*).

The most common raw material in the Nyayanga artifact assemblage, Bukoban quartzite, is absent from the nearest conglomerates. Our survey of radial drainages on the peninsula did not recover a single suitable naturally occurring quartzite cobble from the 2274 total local clasts identified in this study (Fig. 4). The composition of Homa Peninsula conglomerates reported here support previous findings that Bukoban quartzite cobbles are not transported onto the peninsula via fluvial activity (*1*).

Quartzite cobbles are available in drainages east of the peninsula, approximately 13 km away from Nyayanga in conglomerates situated within the reach of the Awach drainage networks (Fig. 4). This is again consistent with previous models of raw material availability, which predict that Bukoban rocks are transported via the Awach rivers but not the Homa Mountain drainages (*1*, *51*, *52*). The nearest of these conglomerates, at the Oboro locality, is located at the distal margins of the Awach Tende basin 13.06 km away. Here, 21 quartzite clasts were observed in the conglomerate (4.4%). Clasts are small overall, with the majority (*n* = 16) measuring under 50 mm in maximum dimension. Quartzite clasts are also present at the Sare-Abururu locality (*n* = 35, 7.0% of conglomerate) in the Lower Awach Kiboun drainage system 18.61 km from Nyayanga.

Nyanzian rhyolite clasts are readily available east of the peninsula around 13 km from Nyayanga, where they are widely emplaced over the region. High frequencies of Nyanzian rhyolite cobbles were present in the Oboro (37.5%) and Sare-Abururu (39.1%) conglomerates. Unfenetized Nyanzian rock was exceptionally rare on the Homa Peninsula. Of 2274 clasts recorded on the peninsula, only two unfenetized Nyanzian rhyolite pebbles were observed, and these were too small to provide suitable blanks for Nyayanga tools. Both clasts measured less than 30 mm in maximum dimension, smaller than any core made from any raw material in the Nyayanga assemblage.

Other materials occasionally present in the Nyayanga assemblage (i.e., chert, 1.7% of assemblage) are found only east of the Homa Peninsula 13 km away. Together, these observations suggest that Nyayanga hominins procured a substantial amount of their lithic raw material from distances of over 10 km.

### Stone transport behavior

**Fig. 6.**
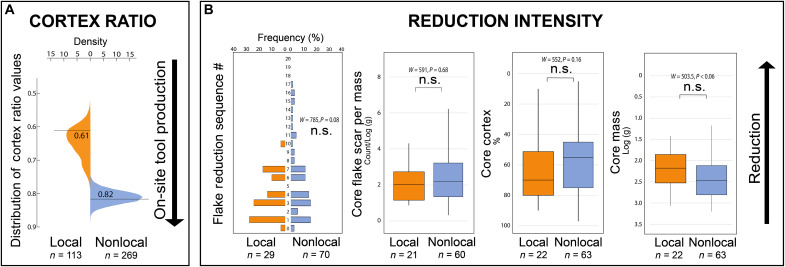
The degree of transport and reduction reflected in local (orange) and nonlocal (blue) artifacts. (**A**) Cortex ratio of local and nonlocal artifacts (solid line). Higher cortex ratio values are indicative of more on-site tool production and discard (Materials and Methods). Artifacts derived from nonlocal raw materials at Nyayanga yield a relatively high cortex ratio (0.82) compared to the cortex ratio of local raw materials (0.61), suggesting that nonlocal lithic resources were not heavily flaked during their transport from their eastern sources to Nyayanga. A density plot reflecting the distribution of possible cortex ratio values in 10,000 Monte Carlo resampling simulations (Materials and Methods) is plotted for local (yellow) and nonlocal (blue) samples. In resampling simulations, 20% of the assemblage is randomly dropped from the assemblage and therefore the width of the distribution (*y* axis) reflects the stability of the observed cortex ratio value. (**B**) Flake reduction sequence number (Materials and Methods) and box and whisker plots displaying core flake scar count/log_10_ (gram), core cortex percent, and core mass in log_10_ (gram) of locally (yellow) and a nonlocally (blue) derived tools in the Nyayanga assemblage. The presence of flakes from later in the reduction sequence and higher ratio of flake scars per core mass suggest more exhaustive reduction. Greater cortex percentage and mass of cores are indicative of less reduction. The *P* values of Wilcoxon-Mann-Whitney tests between local and nonlocal samples are reported. n.s., not significant.

The Nyayanga artifact assemblage deviates from distance-decay expectations, where materials sourced from greater distances are predicted to be more reduced and constitute less mass in the overall assemblage (*35*, *36*). At Nyayanga, nonlocal artifacts make up most of the assemblage mass and exhibit low levels of reduction intensity and high proportions of cortex (i.e., the weathered outer rind), indicating frequent on-site transport and discard within the spatial boundaries of the assemblage. Bukoban quartzite is absent from contemporaneous paleo-conglomerates near the site, yet tools of this material constitute more than half of the total assemblage weight (53.2%; 27,407.8 of 51,541.7 g). Quartzite and other nonlocal flakes are represented at early stages in the reduction sequence, and nonlocal cores underwent similar degrees of reduction compared with local materials (Fig. 6). These data do not support the formation of the Nyayanga artifact assemblage through cumulative unplanned transport bouts, and they contrast with patterns in the data regarding nonhuman primate tool transport (*36*, *37*).

It is possible that hominins could have created caches of manuports at Nyayanga or at nearby penecontemporaneous sites. However, the low weathering stages of NY-1 fossils, discovery of partial hippopotamid skeletons, and well-preserved artifact surfaces are all consistent with the sedimentological interpretation of rapid NY-1 deposition (*50*). It is therefore unlikely that secondary sources of raw material accumulated through hominin activity would have been exposed on the landscape for extended periods of time. Together, these results are consistent with the nonrandom transport of raw materials from eastern sources to Nyayanga, where they were likely reduced and/or used on-site.

Oldowan toolmakers at Nyayanga exhibited similar raw material preference and transport to those known from more recent hominins at Kanjera South (*1*). Hominins at both localities selected durable nonlocal materials, such as Bukoban quartzite and Nyanzian rhyolite, and procured them from kilometers away. Geochemical signatures link Nyayanga rhyolite artifacts to nonlocal sources of Nyanzian rhyolite and local sources of Homa Mountain fenetized rhyolite, but not to Wire Hills rhyolite, another source known in the region (Fig. 5). These are the same local and nonlocal sources of rhyolite that Kanjera South hominins used 600 ka later (*1*). However, at Nyayanga, nonlocal lithic resources were not as heavily flaked and used during their transport from eastern sources as they were at Kanjera South.

## DISCUSSION

The transport of core and flake technology provides evidence for evolving technical and cognitive abilities considered to be essential to the success of genus *Homo*. Yet our understanding of tool transport behaviors at the dawn of the Oldowan Complex remains limited. Stone resource procurement at Nyayanga offers insights into the ranging behaviors and foraging activities of some of the earliest toolmakers.

The Nyayanga lithic assemblage is dominated by a diversity of nonlocal materials unavailable within several kilometers of the locality. The prioritization of nonlocal stones and their long-distance procurement demonstrates a level of raw material transport unobserved in primate tool transport or in other early archaeological assemblages (*15*–*19*, *34*, *39*). Although the taxonomic identity of Nyayanga toolmakers remains unknown, the association of this assemblage with fossils attributed to the genus *Paranthropus* (*50*) calls into question whether the transport of core and flake technology was exclusive to genus *Homo*. Regardless of knowing which hominin(s) produced the artifacts, the differences in raw material transport documented at Nyayanga compared to other early archaeological sites may be best explained by variation in local ecological conditions—particularly the availability of suitable stone raw material locally—or in the niches of the hominins producing Oldowan tools.

The coordinated movement of stone and food resources across landscapes marks an important breakthrough in the evolution of technological behavior. However, determining the source of stone raw materials can be challenging because of geological constraints. On the Homa Peninsula, many conglomerates are estimated to be the same age or older than Nyayanga deposits [(*1*); Table 1], providing the opportunity to study paleo-conglomerate sources that were likely accessible to Nyayanga toolmakers and confirming the absence of Bukoban rocks near Nyayanga. However, identifying contemporaneous Bukoban and Nyanzian sources beyond the peninsula is challenging because Pliocene conglomerates are not exposed on the landscape in the eastern highlands. To address this limitation, past studies have used more recent (i.e., Pleistocene and modern) conglomerates to model the availability of stone materials in earlier periods (*1*, *47*, *53*). These younger conglomerates provide useful analogs for Nyayanga-age conglomerates as their primary sources and drainages remained unchanged since the Pliocene (table S1).

On the basis of the available evidence, stone transport at Nyayanga likely occurred over distances greater than 10 km, similar to those proposed for Kanjera South (*1*). This is supported by a geochemical link between quartzite artifacts from Nyayanga and Bukoban primary sources in the eastern highlands, particularly quartzite found in the Awach Tende drainage basin 13 km away. However, the precise ancient conglomerates that served as sources for these raw materials remain unknown, limiting our ability to calculate an exact transport distance. Furthermore, the lack of Bukoban and Nyanzian rocks in the conglomerates around the Homa Peninsula limits the sample size of our geochemical reference collection. While our rhyolite and quartzite reference sample has been expanded from the previous collections used to source raw materials from the Homa Peninsula (*1*), it remains relatively small (*n* = 55).

At Nyayanga, indicators of core reduction deviate from distance-decay expectations, suggesting that toolmakers transported nonlocal clasts toward the locality, minimally reducing them on the way. The importation of manuports to Nyayanga is consistent with foraging strategies that emerge from the anticipation of food processing needs (*57*). Hominins may have delayed or minimized the within-journey use of cobbles when carrying them from eastern sources, anticipating plant and animal resource processing tasks on the Homa Peninsula (*50*). The apparent landscape-scale mental maps of Nyayanga hominins imply knowledge of not only the distribution and quality of local and non-local stone but also of the distribution of organic resources requiring tool-assisted processing and how those resources could be most beneficially exploited. The presence of flakes and cores at various stages of reduction (Fig. 6) and evidence of on-site tool production (e.g., hammerstones and cortical flakes) support the conclusion that Nyayanga was a place where toolmakers imported stone, produced tools, and returned repeatedly.

Nyayanga artifacts were deposited along the margins of a large stream in a C_4_ grazer–dominated ecosystem, in proximity to trees and a freshwater spring (*50*). The recovery of hominin fossils alongside evidence of plant and animal processing with stone tools, including the earliest documented megafaunal butchery, support the view that the Nyayanga locale was an attractive setting for hominin activities. Bukoban quartzite and other hard, nonlocal raw materials were transported from the eastern drainages because the available lithologies at Nyayanga were less durable and unsuited to the sustained plant and animal processing indicated by lithic use-wear (*50*). The frequency of pounding damage on the Nyayanga artifacts is high for an Oldowan assemblage (*50*). Many pounding tools are large quartzite cobbles, suggesting that nonlocal clasts were sometimes transported to Nyayanga specifically for pounding tasks, in addition to producing flakes for cutting and scraping activities.

The emphasis on pounding tools at Nyayanga suggests that their use-life conditions are more similar to those of nonhuman primate percussive technology than most Oldowan assemblages that were primarily focused on producing flakes for cutting tasks. Despite this similarity, the transport pattern observed at Nyayanga substantially differs from the raw material procurement strategies of nonhuman primates. The earliest Oldowan toolmakers foraged for stones to use as percussive tools over much longer distances than is typically observed in primate percussive technology, indicating that Oldowan toolmakers deviated from nonhuman primate stone transport, even when procuring pounding tools.

Dissimilar patterns of raw material transport may arise at different localities due to differences in (i) local ecological settings, (ii) the distribution of stone and food resources, and (iii) the hominin taxa involved. One explanation for why other early Oldowan sites do not preserve evidence of complex transport dynamics is that they were situated near channel conglomerates with abundant high-quality raw materials [i.e., phonolite at West Turkana, basalts at Gona and Hadar, trachyte at Gona, and rhyolite at Hadar and Gona (*15*–*17*)]. Mechanical tests indicate that the lithologies available in local conglomerates on the Homa Peninsula were markedly less durable than the nonlocal lithologies (*47*) located to the east and south, which likely factored into the longer-distance transport of more durable raw materials to Nyayanga. We hypothesize that the degree of spatial disparity in the distribution of stone versus food resources may explain much of the variability in Oldowan hominin stone transport behavior.

It is also possible that variable resource transport strategies reflect differences in the ecological niches and ranging behaviors of the toolmakers. Fossil evidence supports that members of both the *Paranthropus* and *Homo* genera are found in spatial association with Oldowan tools (*58*, *59*), raising the possibility that both taxa may have used this technology, potentially deriving this behavior from a shared, stone tool–using ancestor. If multiple hominin taxa engaged in Oldowan toolmaking, this could explain some of the variation observed in raw material procurement patterns across contemporaneous assemblages. At Nyayanga, it is possible that toolmakers had different diets and foraging strategies compared to the hominins producing other assemblages.

The Nyayanga data indicate that by at least 2.6 Ma, the technological repertoire of hominin toolmakers included the ability to bypass local materials in favor of higher-quality stones, even when this required transport over long distances. This is similar to patterns of stone resource movement at Kanjera South (*1*, *60*), suggesting that comparable lithic transport occurred on the Homa Peninsula at multiple localities and points in time at least 600,000 years apart. Moving stones across the landscape was not an isolated event and may have been typical for Oldowan hominins inhabiting areas where attractive resources requiring stone tool processing and high-quality raw materials were distant from each other. The Nyayanga evidence suggests that late Pliocene hominins transported stone resources over kilometers as part of a broad, landscape-wide foraging strategy distinct from the use and re-use patterns that characterize non-human primate stone tool behavior. The transport of nonlocal materials at Nyayanga demonstrates that the earliest Oldowan toolmakers used landscape-scale knowledge to unite spatially disparate resources, providing evidence of a developing system of anticipatory behavior and a foraging strategy unique to hominins.

## MATERIALS AND METHODS

### Nyayanga, Kenya

The Homa Peninsula is situated in the Nyanza rift area of Kenya between the two branches of the East African Rift System, on the southern margin of the Kavirondo Gulf of Lake Victoria (Fig. 2). Nyayanga is located on the Homa Peninsula on the western flank of the Homa Mountain carbonatite complex. This archaeological and paleontological locality preserves one of the oldest Oldowan artifact accumulations (3.040 to 2.581 Ma) (*50*). From 2016 to 2023, the Homa Peninsula Paleoanthropological Project conducted two excavations yielding 129 in situ artifacts and 272 artifacts collected on the surface of bed NY-1.

We determined the raw material composition of the Nyayanga artifact assemblage and 11 secondary drainages (conglomerates) around the Homa Peninsula, Kenya. Raw materials were identified using a combination of geochemical characterization and visual inspection with a 10× hand lens. These results were then compared with data from previous work that characterized the geochemistry and composition of the Kanjera South assemblage and other nearby primary and secondary sources (*1*, *53*). The details of the conglomerate survey, geochemical methods, and technological analysis are described below.

### Conglomerate sample

Previous geological survey has provided models of raw material availability of the Homa Peninsula and surrounding area (*1*, *51*, *52*). However, these surveys focused on the northern half of the peninsula, while Nyayanga is located on the peninsula’s western margin. Our goal was to determine broad patterns of lithological availability around the peninsula, with a focus on the west and south. Eleven conglomerates were selected for analysis (fig. S1). A random sample of cobbles (mean, *N* = 361; SD, 122.4; range = 149 to 500) were collected from a 1- to 2-m^2^ section of each conglomerate (figs. S2 and S3). Clasts were sorted by size, angularity, and raw material type. Cortical pieces were fractured using a rock hammer to determine the lithology.

The conglomerates nearest Nyayanga are from a contemporaneous drainage system that can be traced through the same unit (NY-1) as the archaeological assemblage. The other conglomerates surveyed in this study are preserved within Plio-Pleistocene deposits, with the exception of one modern conglomerate (Table 1). Many conglomerates occur in units that correlate with the Nyayanga stratigraphic sequence.

Previous survey of the NY-1 equivalent conglomerate (*53*) confirmed the absence of Bukoban rock. However, two unaltered Nyanzian rhyolite pebbles were identified at the Dago locality, less than 2 km from Nyayanga. On the basis of this finding, it was proposed that the Dago conglomerate may have provided a potential source of unfenetized rhyolite (*53*). However, further study at Dago suggests that this is unlikely as no additional unfenetized Nyanzian rhyolite clasts have been found at the site, and the two previously reported (*53*) are not suitable blanks for tools. These two Nyanzian pebbles are reported and discussed again in our current study.

At Nyayanga, the Homa Formation (Homa Fm) lays unconformably at the base of the sequence. The Homa Fm is a stratigraphically complex sequence that has been investigated most extensively at Kanam West and is estimated to have been deposited ~4 to 3.5 Ma based on biostratigraphy (*61*, *62*). The Homa Fm outcrops in many locations around the Homa Peninsula often in areas where more recent stratigraphic beds are also exposed.

At Luanda West and Homa Lime, the conglomerates are situated in the Homa Formation. These conglomerates were formed by fluvial activity coming from the Homa Mountain radial drainage system that would have predated the accumulation of the Nyayanga artifact assemblage. These sources of stone would have existed and likely been accessible to hominins forming the NY-1 archaeological assemblage.

At Homa Lime, the Homa Fm forms the apparent base of a sequence overlain unconformably with more recent deposits containing stone tools. The artifact-yielding upper units appear to be Pleistocene in age and contain mode 1 lithics. Four quartzite clasts were recovered from the Homa Lime locality and previously reported as a possible source of quartzite on the peninsula (*53*). However, on further inspection in this current study, three of these clasts preserve evidence of pounding damage and/or flake removals and are classified as archaeological. These previously reported quartzite clasts likely derive from the younger Pleistocene deposits yielding stone-age artifacts that deflated onto the surface of the older conglomerate.

At Luanda West, the Homa Fm underlays a 5-m sequence of fluvially deposited pebbly sands and silts exposed in gulley beds. A volcanic tuff previously identified as the Wakondo tuff dated to ~100 ka (*63*, *64*) is traceable over much of the gulley and fossils and artifacts characteristic of the Middle Stone Age are preserved on the surface. The artifact assemblage is characterized by small cores and flakes including discoidal cores, points, and blades.

The conglomerates at the Bala and Kananga locality also outcrop in deposits resembling the Homa Fm. However, more work is necessary to definitively identify the stratigraphic sequence at these localities. The presence of mode 1 artifacts at Bala suggests an early Pleistocene age. At Kananga, artifacts are similar to Middle Stone Age technologies documented at Luanda West.

Stratigraphic beds at Onyango and Komullo visually resemble the NY-3 and NY-4 units at the Nyayanga locality. However, additional work would be necessary to determine whether these units correlate. The conglomerates at Sare-Abururu, Kisaka, and Oboro are younger in age than the Nyayanga sequence. They range from ~1.7 Ma [Sare-Abururu (*65*)], to modern (Oboro). At the Kisaka locality, Middle Stone Age artifacts are exposed on the surface of a gully, and a volcanic tuff is visible. The exposed tuff may represent the Wakondo Tuff (~100 Ka), which is known to outcrop widely through western Kenya (*64*). These particular conglomerates would not have been available to Nyayanga hominins. However, their composition represents regional patterns of raw material availability that would have remained relatively unchanged through the Plio-Pleistocene. The primary sources of Homa, Bukoban, and Nyanzian raw materials are ancient, and regional drainages followed the same regional patterns over time because the topography of the Homa Mountain and the regional faults that govern the eastern drainage systems have been in place since the early Pliocene (*51*–*53*).

### Geological characterization

All artifact (*n* = 401) and conglomerate rock samples (*n* = 3833) were attributed to lithological groups through visual inspection with a 10× hand lens. A subset of artifacts (*n* = 316) and geological samples (*n* = 55) was then subject to nondestructive ED-XRF analysis and trace element concentrations were obtained. The lithics subject to ED-XRF included all Nyayanga artifacts of any raw material that were collected before 2022 and suitable for analysis (more than 20 mm and small enough to fit in a 171.45 mm–by–101.6 mm sample chamber). This added up to 316 artifacts (79% of the total assemblage). Artifacts from excavation 3 (*n* = 112), excavation 5 (*n* = 14), and surface artifacts (*n* = 190).

Five tools were excluded because they were too large to fit in the sample chamber, and two artifacts were excluded because they were too small to cover the sample screen. The constraint of the machine introduces potential bias by omitting both large and small artifacts from the analysis. However, only seven artifacts were excluded because of size and all were nonlocal. Of the five tools too large for analysis, all originated from nonlocal rock types and the majority were quartzite (*n* = 3). The small artifacts omitted were debitage made from quartz (*n* = 1) and Bukoban quartzite (*n* = 1).

The majority of artifacts excluded from analysis were omitted because they were recovered after 2022 (*n* = 71). The post-2022 artifacts broadly reflect the Nyayanga artifact assemblage. The artifacts are fenetized rhyolite (*n* = 13), Nyanzian rhyolite (*n* = 5), Bukoban quartzite (*n* = 36), quartz (*n* = 11), Bukoban felsite (*4*), fenetized andesite (*1*), and Homa phonolite (*1*). Additional five artifacts were excluded for miscellaneous reasons (i.e., there were errors in the analysis).

The trace geochemistry of all the artifacts (*n* = 316) subjected to ED-XRF are reported in auxiliary table S1. Of the 316 samples analyzed, the artifacts manufactured from quartzite (*n* = 86) and fenetized rhyolite (*n* = 81) and Nyanzian rhyolite (*n* = 35) provided the most useful data for determining transport distance and distinguishing between sources.

### Geochemical techniques

Geochemical fingerprinting using XRF is useful for differentiating raw materials sources in the Early Stone Age (*66*). Previous research has linked Kanjera South artifacts to their closest known primary (outcrop) and secondary (drainage conglomerate) sources using geochemical signatures (*8*, *51*, *53*). The ratio of strontium to zirconium is especially informative when distinguishing between sources of rhyolite around the Homa Peninsula (*8*, *53*). We chose to source using trace element geochemistry rather than silica values because the nature of the artifact assemblage requires nondestructive techniques.

The protocol for determining elemental concentrations is described in detail in Finestone *et al.* (*53*). The concentration of the elements rubidium (Rb), strontium (Sr), yttrium (Y), and zirconium (Zr) were estimated using an Amptek Experimenter’s XRF Kit housed at the University of Nairobi Institute of Nuclear Science and Technology. Three faces of each sample were exposed to the primary x-ray beam, and trace element concentrations from the three surfaces were averaged. This technique reduces the effects of surface heterogeneity for rhyolite and quartzite artifacts and results in lower relative SDs between repeated trials of the same specimen (*53*). The average relative SDs observed between three faces of the same artifact ranged from 20 to 38% across the elements of interest (table S2). Samples were placed on a sample holder and irradiated for 200 s with the instrument set at a voltage of 30 Kev and a current of 80 μA. The spectra were collected using Amptek DppMCA Display and Acquisition software. Deconvolution of the spectral data was performed using AXIL software. Elemental concentrations were calculated using the semiquantitative analysis method of direct comparison, which relates the areas of the respective peaks to concentration values in parts per million. The instrument detection limit for each element was calculated on the basis of the background count rate, the concentration, and the peak area of each element. If a samples average element concentration was less than the instrument’s detection limits (table S3), the elemental value was omitted from the analysis.

Elemental concentrations were calibrated from USGS reference standards W-2, BHVO-1, SCO-1, BIR-1 and RGM-1 (table S4). The efficiency calibration curve was generated by plotting the intensities of the characteristic elements against respective concentration values using the direct comparison method of the AXIL program. The resulting calibration curve was then used in the quantification of Rb, Sr, Y and Zr. Two standards, BHVO-1 and BIR-1, were run as unknowns following calibration with the W-2, SCO-1, and RGM-1 standards to evaluate the accuracy of elemental values in this experimental setup. Observed values for elements Rb, Sr, Y, and Zr fell within the range of recommended values, or, with exception of the Sr concentration in BHVO-1, differed by only several parts per million. The measured value fell 20 ppm outside of the recommended value (table S5).

Braun *et al.* (*1*) previously undertook an ED-XRF study of archaeological material from the Oldowan locality Kanjera South (*54*) and obtained semiquantitative concentrations of Rb, Sr, Y, and Zr for artifacts and primary and secondary source samples representing a variety of lithologies around the northern half of the Homa Peninsula. The elemental compositions determined for Nyayanga artifacts and clasts from conglomerates were calibrated and transformed according to Finestone *et al.* (*53*). The resulting dataset was compared to the XRF data collected by Braun *et al.* (*1*) for provenancing the Kanjera South artifact sample.

We compared artifact and source samples using bivariate plots of element concentration in parts per million logarithmically transformed. The bivariate plots captured separation between the types of raw material and sources, making it unnecessary to apply multivariate approaches such as linear discriminant analysis or principal components analysis.

### Technological measurements

Artifacts were divided into categories of detached pieces (i.e., debitage), percussion pieces [(*67*) i.e., hammerstones], flaked pieces (i.e., cores), or manuports. Detached pieces were further classified into debitage types (whole flakes, snapped flakes, split flakes, split and snapped flakes, or angular fragments). Each artifact was weighed, and maximum dimension was recorded. Maximum dimension was measured in millimeters as the longest distance across an artifact. Mass was measured with a digital scale to the 10th of a gram. Measures of reduction intensity in the Nyayanga artifact assemblage were estimated from the features of both flakes and cores.

#### Flake attributes

In addition to maximum dimension, flake length, width, and thickness were measured in millimeters with digital calipers. Length was defined as the distance between the point of initiation to the distal end of the flake on the ventral surface in the direction of percussion. Width was measured as the distance across the ventral surface of the flake at the midpoint, perpendicular to the axis of technological length. Thickness was measured at the axis perpendicular to width and length.

Attributes of the dorsal surface, such as cortex and characterization of flake scars, were measured. Flake scars are the negative impressions made from previous removals with a maximum dimension greater than 10 mm. The number, direction, and termination of dorsal flake scars were recorded. Dorsal cortex is an estimation of the percentage of cortex on the dorsal surface. Here, it was estimated using an eight-stage system previously used by Roth and Dibble (*68*) and Braun *et al.* (*69*). This system assigns a number 1 to 8 based on the degree of remnant cortex present (8 = 0%; 7 = 1 to 10%; 6 = 11 to 30%; 5 = 31 to 50%; 4 = 51 to 70%; 3 = 71 to 90%; 2 = 91 to 99%; and 1 = 100%). The termination type of flake scars was also indicated as feather, step, hinge, or overshoot.

The number of platform facets and amount of platform cortex were also recorded. Platform cortex was determined in 10% intervals (e.g., 0, 1 to 10%, 10 to 20%, etc.). Low percentages of cortex (both on flake dorsal surfaces and platforms) indicate a lack of representation of initial flakes stages. Characterizing the number and direction of flake scars on the dorsal surface of flakes and cores groups artifacts according to exploitation category and tells us about the reduction strategy.

#### Reconstructing flake reduction sequence order

Grouping flakes according to Technological Flake Categories (*70*–*72*) is heavily influenced by initial clast size such that larger cores are significantly associated with higher frequencies of flake types 1 to 3 (*69*, *73*). An alternate method for reconstructing reduction sequence in Oldowan assemblages is Braun’s multiple linear regression model that incorporates several other variables to more accurately predict a flake’s position in the reduction sequence (*69*). Braun *et al.* (*69*) found that the best predictor of flake sequence took into account the number of dorsal flake scars, the degree of dorsal cortex (in the eight-stage system), the number of dorsal flake scar directions (where differences in directions over 30° were considered different directions), and technological length and width. The formula for the stepwise multiple linear regression model with the lowest SE of estimate applicable to Oldowan flaking was

Flake sequence number = [(flake scar direction × 0.628) + (platform facets × 1.008)] + [flake scar/log (flake length × flake width) × 0.159] + [dorsal cortex/log (flake length × flake width) × 0.097]^2^

This formula was used to reconstruct a flake’s stage in the reduction sequence.

#### Core attributes

The maximum length, width, and thickness of each core was measured using a digital caliper in millimeters. Core length was defined as the longest axis of the cobble. Core width was measured perpendicular to the axis of maximum length. Core thickness was defined as the maximum dimension that is perpendicular to the axis of length and the axis of width. Core percent cortex was an estimation of the amount of cortex covering the whole piece and was estimated within 5%. The number of flake scars on the core were also recorded. Only flake scars greater than 20 mm on the surface of the core were counted.

### Assemblage cortex ratio

As a clast is flaked, cortex is removed. The proportion of cortex observed in an assemblage provides a measure of on-site reduction and discard. Cortex ratio is an analytical method that is calculated by dividing the amount of cortex observed in the assemblage by the expected amount. This estimate is expressed as a ratio of the cortex present relative to what is expected if the nodules responsible for the assemblage were reduced and discarded entirely within the sample (*74*–*78*). Therefore, a cortex ratio of 1 will occur if the amount of cortex in the sample is equal to the amount of cortex that would have been present on the original nodules responsible for producing the assemblage. Cortex ratios closer to 1 reflect on-site production and use, where most cobbles are knapped, used, and discarded within the spatial confines of the assemblage (*76*). Deviations from cortex ratios of 1 imply that stone tools were manufactured across a greater area than the scale of the assemblage. This can mean that nodules were either used and discarded before their arrival at the site or that some artifacts were transported away from the site. Thus, departures from 1 indicate a degree of stone tool transport. In this study, both the observed amount of cortex and the expected amount of cortex were calculated according to Douglass *et al.* (*75*).

#### Calculating observed cortex in assemblage

To calculate the observed amount of cortex, the amount of cortical surface area was estimated for each artifact in the assemblage. Estimating surface area: First, the total surface area was calculated for each artifact. For detached pieces, the surface area was estimated by multiplying the maximum length of the artifact by its maximum width. Surface area for cores, pounded pieces, and manuports were estimated by using the semi-axes of each artifact to calculate the surface area of scalene ellipsoid (*76*).

Estimating cortical surface area: Cortex on each artifact was then classified into intervals of 5%. Although these measures are relatively coarse, work comparing more detailed classifications with actual measurements of the cortical surface area show that estimates based on naked eye observations are most accurate (*79*). This proportion of cortex was then multiplied by the total artifact surface area to estimate cortical surface area of each artifact. The cortical surface area of each artifact was then summed to get the observed amount of cortex within the assemblage.

#### Calculating expected cortex in assemblage

Calculating the expected amount of cortex requires estimating the number of nodules required to produce the assemblage of a given size, their average size, and the original surface area. Here, we assume that the number of nodules required to produce the assemblage is equal to the number of cores, pounded pieces, and manuports present. The mean original nodule size is then calculated by dividing the total volume of the assemblage by the number of cores, pounded pieces, and manuports. The surface area of the average original nodule is calculated using the equation for the surface area of a scalene ellipsoid. A scalene ellipsoid was chosen as it most similarly resembles the river cobbles used by Early Stone Age hominins on the Homa Peninsula. The expected amount of cortical surface area is then estimated by multiplying the average nodule surface area by the number of cores within the assemblage.

#### Measuring cortex ratio stability

Because the cortex ratio is effectively a surface area to mass ratio, some large objects have more influence on the calculation than smaller artifacts. When sample sizes are low, this can cause the cortex ratio to be reflective of a few larger pieces as opposed to being representative of the entire assemblage. To ensure that the cortex ratio reflects assemblage scale tool transport, we use a Monte Carlo resampling scheme to assess the stability of observed values at Nyayanga. In this resampling simulation, 20% of the assemblage is randomly dropped from the assemblage. The cortex ratio is then recalculated on this reduced assemblage. This process carried out for 10,000 iterations for each raw material class (local and nonlocal) to generate a distribution of possible cortex ratio values. The width of this distribution provides a measure of the level of uncertainty surrounding each observed cortex ratio value.

### Curation and research ethics

This project was designed and carried out in collaboration with coauthors from the University of Nairobi and from the National Museums of Kenya. Cultural artifacts are curated at the National Museums of Kenya in Nairobi, and laboratory analysis was undertaken by researchers at the University of Nairobi Institute of Nuclear Science and Technology. No scientific samples were transferred out of the country of Kenya to undertake this project. Permission for this research was granted by the Kenya Government Ministry of Sports, Culture and the Arts (NACOSTI permit #P/14/7709/701).

### Statistical analysis

We performed a two-sample *t* test to compare the mass of quartzite tools compared to the rest of the artifact assemblage. Wilcoxon-Mann-Whitney tests were performed to compare the difference between artifacts manufactured on local and nonlocal raw materials in terms of their flake reduction sequence number, core flake scar count per mass, core cortex, and core mass. For all statistical tests, significant differences were determined using an alpha level of 0.05 and all statistical tests were performed in R v. 4.2.2 (*80*).
