# Supplementary material for: Selective use of distant stone resources by the earliest Oldowan toolmakers
Source: Sci Adv. 2025 Aug 15;11(33):eadu5838. doi: 10.1126/sciadv.adu5838 (PMC12356259; doi:10.1126/sciadv.adu5838)
Supplement: Supplementary file 1 — Supplementary Text Figs. S1 to S7 Tables S1 to S5 Legends for data files S1 to S4 [file sciadv.adu5838_sm.pdf]

Science Advances
AAAS

Supplementary Materials for

Selective use of distant stone resources by the earliest Oldowan toolmakers

Emma M. Finestone *et al.*

Corresponding author: Emma M. Finestone, efinestone@cmnh.org

Sci. Adv. **11**, eadu5838 (2025)
DOI: 10.1126/sciadv.adu5838

The PDF file includes:

Supplementary Text
Figs. S1 to S7
Tables S1 to S5
Legends for data files S1 to S4

Other Supplementary Material for this manuscript includes the following:

Data files S1 to S4

Provenance

Nyayanga (0° 23.909'S, 34° 27.115'E) was first surveyed by the Homa Peninsula Paleoanthropological Project, a collaboration between researchers at the National Museums of Kenya, the City University of New York, and the Smithsonian Institution, in 2000. Geological observations and surface collections were carried out in brief visits in 2007, 2011, 2012, and 2014. Excavations 1 and 2 were carried out in 2015 but recovered little. Excavations 3-6 were initiated in 2015, and all yielded fossils and stone tools (50). Sediments were excavated in 5 cm spits in 1 m squares using scratch awls and dental picks, or with a scratch awl struck with a light hammer when sediments were hard. All potentially identifiable remains <2 cm in length and all remains >2 cm in length were individually plotted in three dimensions using a laser theodolite. A unique field number was assigned each specimen and its surrounding geological bed and sediment described. Excavations and surface collection focused on the top half of the oldest bed (NY-1) which yielded Oldowan artifacts, *Paranthropus* sp. fossils, and faunal fossils in overbank deposits from a westward-flowing paleochannel (50).

The age of the NY-1 Beds is constrained by (U-Th)/He dating of apatite crystals, magnetostratigraphy, lithostratigraphic correlation with the Rawi Formation deposited north of Homa Mountain, and biostratigraphy (50). All lines of evidence suggest that lower NY-1 deposition occurred in the temporal range of the C2An.1n Subchron between 3.032 and 2.595 Ma (50).

All fossils and artifacts collected by the Homa Peninsula Paleoanthropological Project are curated in the collections of the National Museums of Kenya in Nairobi.

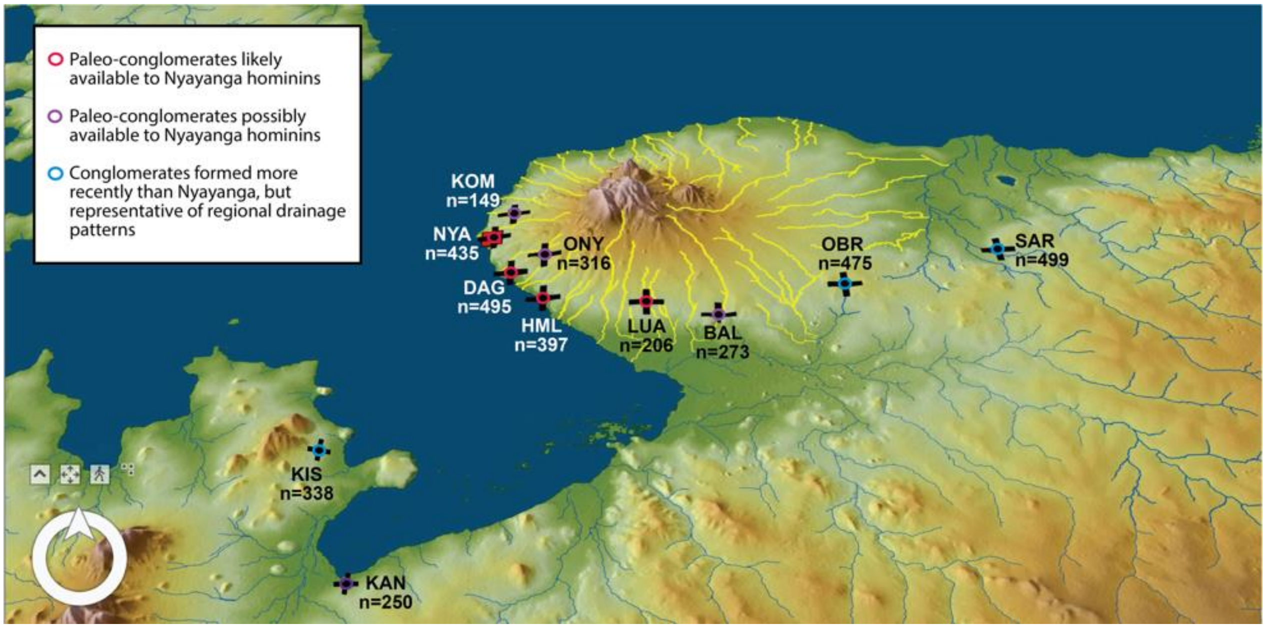

Fig S1. A map of the conglomerates surveyed in this study with sample size and predicted availability to the Nyayanga hominins. NYA = Nyayanga, HML = Homa Lime, BAL = Bala, DAG = Dago, SAR = Sare Abururu, LUA = Luanda West, OBR = Oboro, KOM = Komullo, ONY = Onyango, KIS = Kisaka, KAN = Kananga.

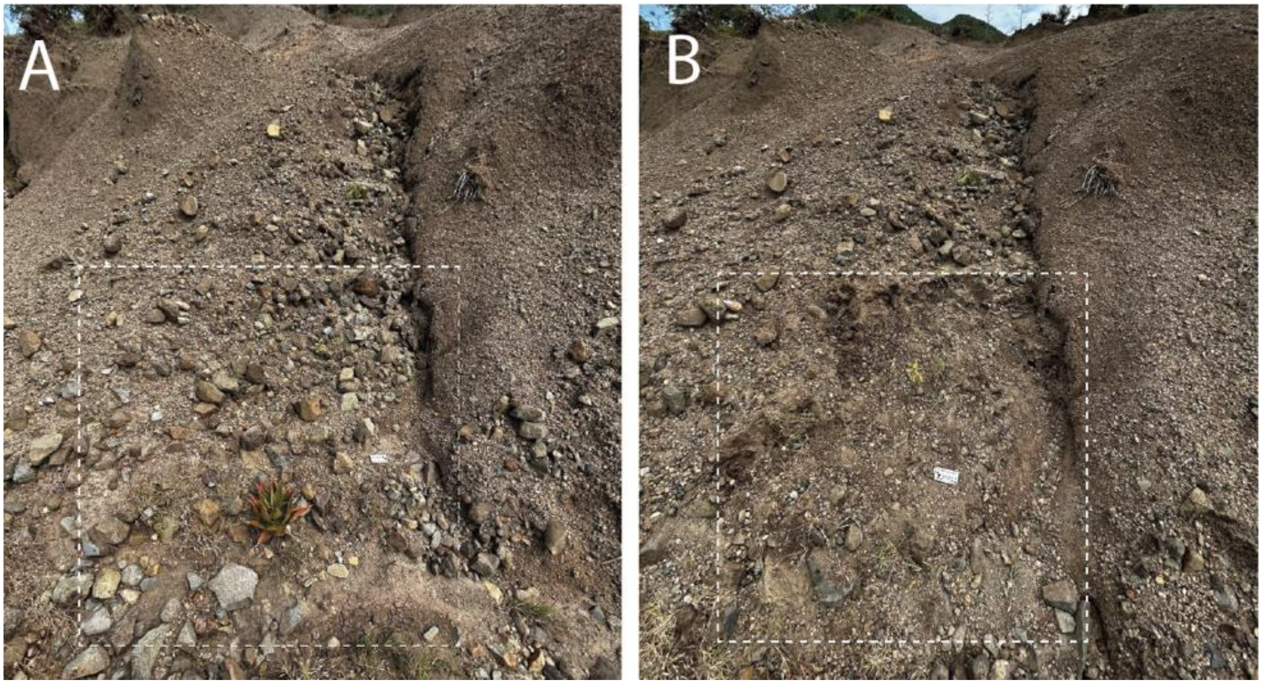

Fig. S2. The sampling area from the Kisaka conglomerate. All clasts were collected from an approximate 1.5 x 1.5 meter area. (A) The area before sampling. (B) The area after sampling. (Photo Credit: Emma Finestone, Cleveland Museum of Natural History).

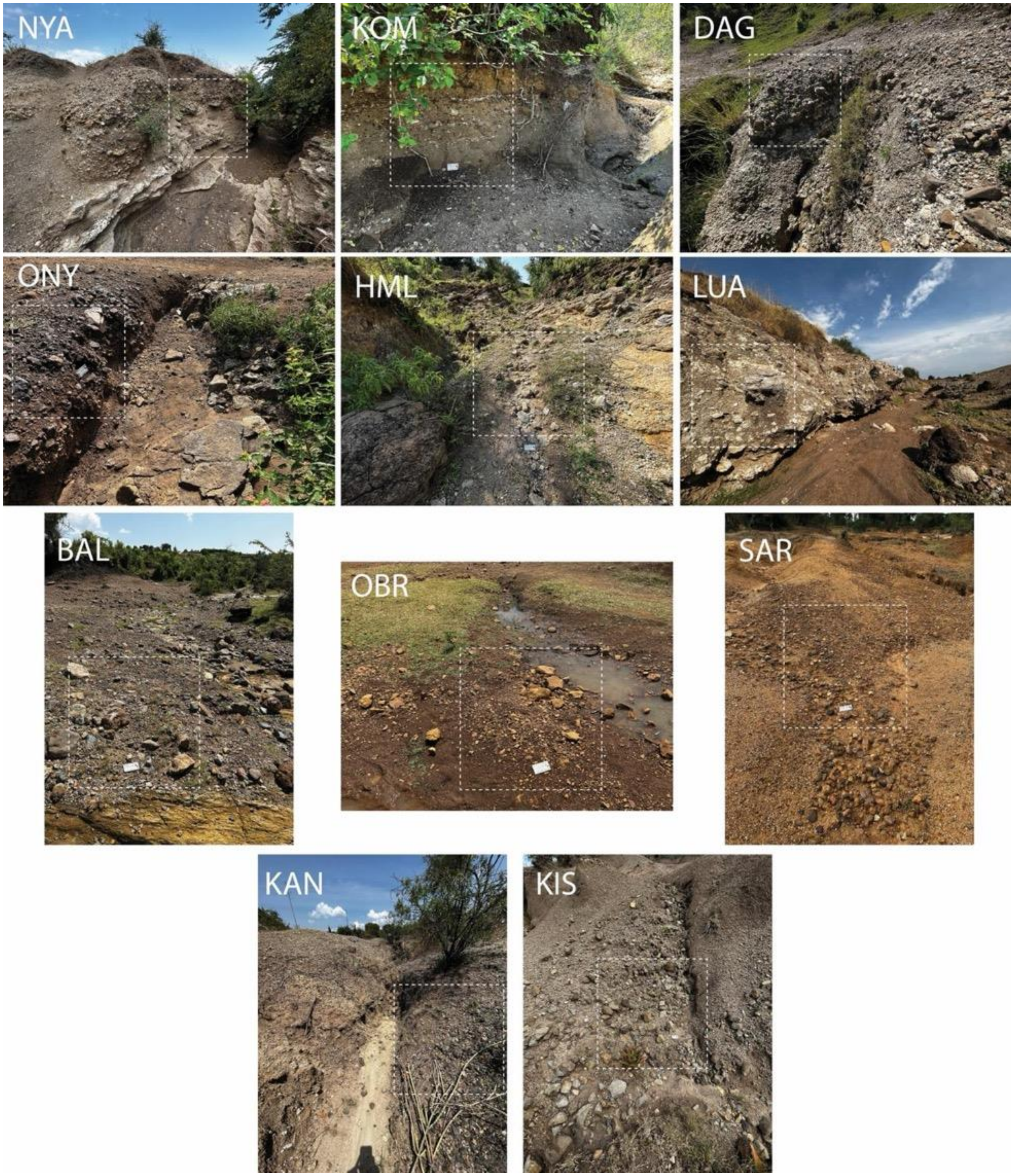

Fig. S3. The sampling area from all conglomerates surveyed in this study. NYA = Nyayanga, HML = Homa Lime, BAL = Bala, DAG = Dago, SAR = Sare Abururu, LUA = Luanda West, OBR = Oboro, KOM = Komullo, ONY = Onyango, KIS = Kisaka, KAN = Kananga. (Photo Credit: Emma Finestone, Cleveland Museum of Natural History).

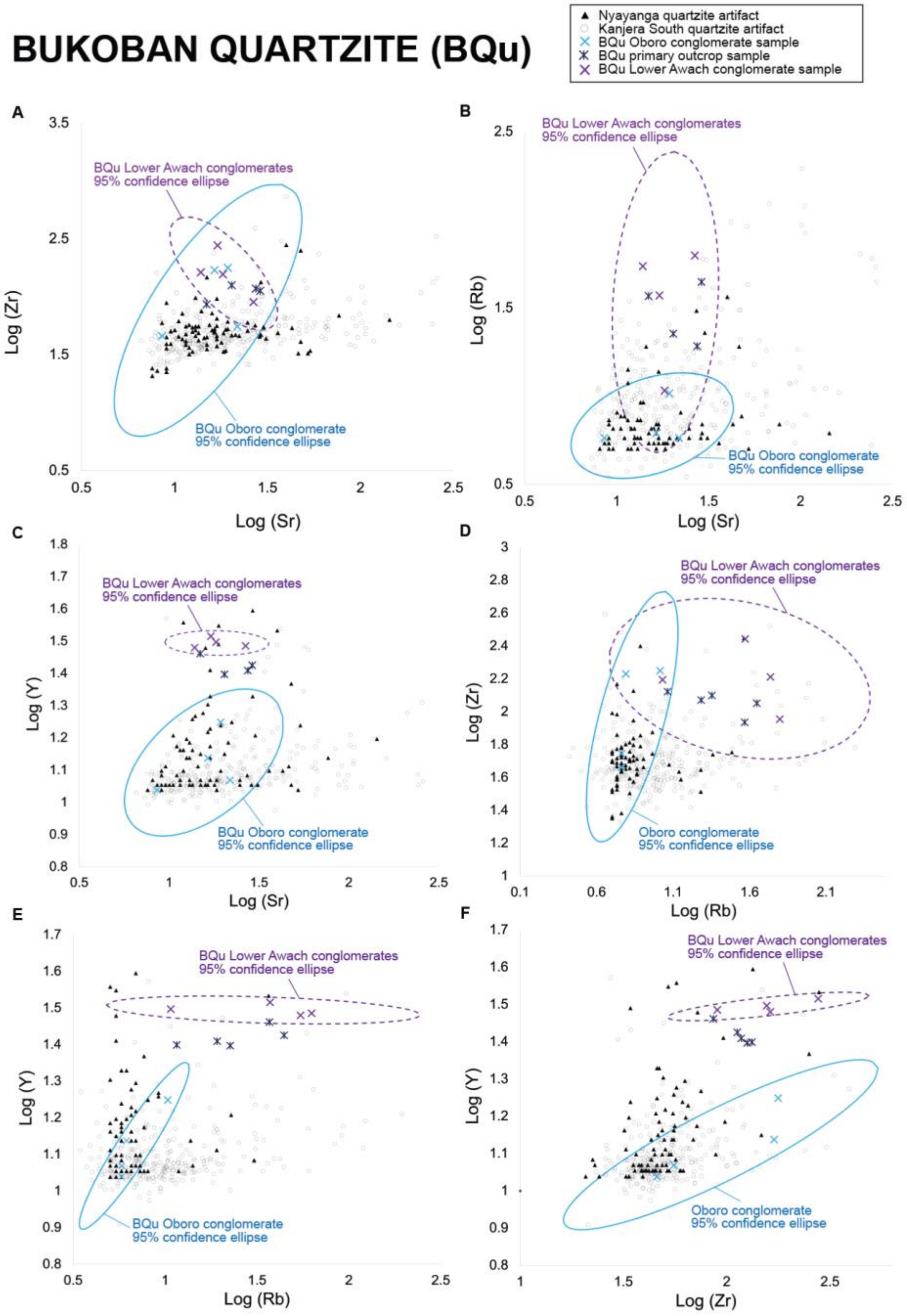

Fig. S4. Bivariate plots of rubidium (Rb), strontium (Sr), yttrium (Y), and zirconium (Zr) logarithmically transformed concentrations for the Bukoban quartzite sample. The geochemistry of Bukoban quartzite (BQu) primary sources (dark blue), Lower Awach Kiboun secondary conglomerates identified by Braun et al. (1) (purple), and Oboro conglomerate quartzite (light blue) are plotted alongside quartzite artifacts from Nyayanga (triangles) and Kanjera South (open circles). 95% confidence intervals for the Lower Awach Kiboun and Oboro quartzite conglomerates are overlain. A. The ratio of $\log_{10}$ (Sr) (x-axis) and $\log_{10}$ (Zr) (y-axis) concentrations in parts per million (ppm) for all artifacts attributed to quartzite from the Nyayanga (n = 82) and Kanjera South (n = 290) assemblages. B. The ratio of $\log_{10}$ (Sr) (x-axis) and $\log_{10}$ (Rb) (y-axis) concentrations in parts per million (ppm) for all artifacts attributed to quartzite from the Nyayanga (n = 75) and Kanjera South (n = 285) assemblages. C. The ratio of $\log_{10}$ (Sr) (x-axis) and $\log_{10}$ (Y) (y-axis) concentrations in parts per million (ppm) for all artifacts attributed to quartzite from the Nyayanga (n = 85) and Kanjera South (n = 289) assemblages. D. The ratio of $\log_{10}$ (Rb) (x-axis) and $\log_{10}$ (Zr) (y-axis) concentrations in parts per million (ppm) for all artifacts attributed to quartzite from the Nyayanga (n = 75) and Kanjera South (n = 285) assemblages. E. The ratio of $\log_{10}$ (Rb) (x-axis) and $\log_{10}$ (Y) (y-axis) concentrations in parts per million (ppm) for all artifacts attributed to quartzite from the Nyayanga (n = 75) and Kanjera South (n = 284) assemblages. F. The ratio of $\log_{10}$ (Zr) (x-axis) and $\log_{10}$ (Y) (y-axis) concentrations in parts per million (ppm) for all artifacts attributed to quartzite from the Nyayanga (n = 82) and Kanjera South (n = 289) assemblages.

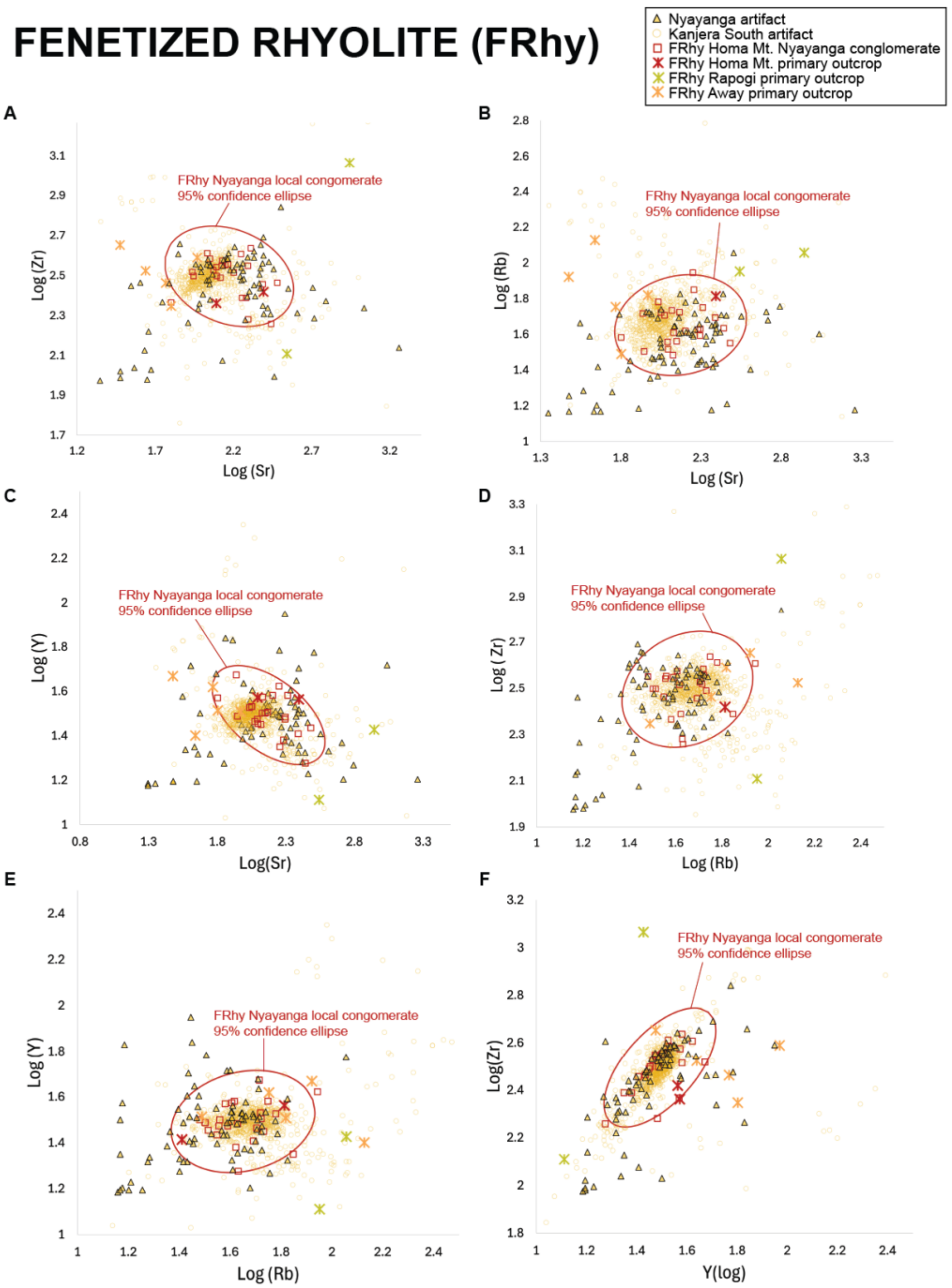

Fig. S5. Bivariate plots of rubidium (Rb), strontium (Sr), yttrium (Y), and zirconium (Zr) logarithmically transformed concentrations for fenetized Nyanzian rhyolite. The geochemistry of fenetized Nyanzian artifacts from Nyayanga (triangles) and Kanjera South (open circles) are plotted with Homa Mt. (red), Rapogi (yellow), and Awaya Hills (orange) primary outcrops of fenetized rhyolite (FRhy). A 95% confidence ellipse for Homa Mountain (Homa Mt.) secondary sources of fenetized rhyolite from the Nyayanga local conglomerate (red boxed) is overlaid. A. The ratio of $\log_{10}$ (Sr) (x-axis) and $\log_{10}$ (Zr) (y-axis) concentrations in parts per million (ppm) for all artifacts attributed to quartzite from the Nyayanga (n = 78) and Kanjera South (n = 755) assemblages. B. The ratio of $\log_{10}$ (Sr) (x-axis) and $\log_{10}$ (Rb) (y-axis) concentrations in parts per million (ppm) for all artifacts attributed to quartzite from the Nyayanga (n = 78) and Kanjera South (n = 755) assemblages. C. The ratio of $\log_{10}$ (Sr) (x-axis) and $\log_{10}$ (Y) (y-axis) concentrations in parts per million (ppm) for all artifacts attributed to quartzite from the Nyayanga (n = 81) and Kanjera South (n = 755) assemblages. D. The ratio of $\log_{10}$ (Rb) (x-axis) and $\log_{10}$ (Zr) (y-axis) concentrations in parts per million (ppm) for all artifacts attributed to quartzite from the Nyayanga (n = 78) and Kanjera South (n = 755) assemblages. E. The ratio of $\log_{10}$ (Rb) (x-axis) and $\log_{10}$ (Y) (y-axis) concentrations in parts per million (ppm) for all artifacts attributed to quartzite from the Nyayanga (n = 78) and Kanjera South (n = 755) assemblages. F. The ratio of $\log_{10}$ (Y) (x-axis) and $\log_{10}$ (Zr) (y-axis) concentrations in parts per million (ppm) for all artifacts attributed to quartzite from the Nyayanga (n = 78) and Kanjera South (n = 755) assemblages.

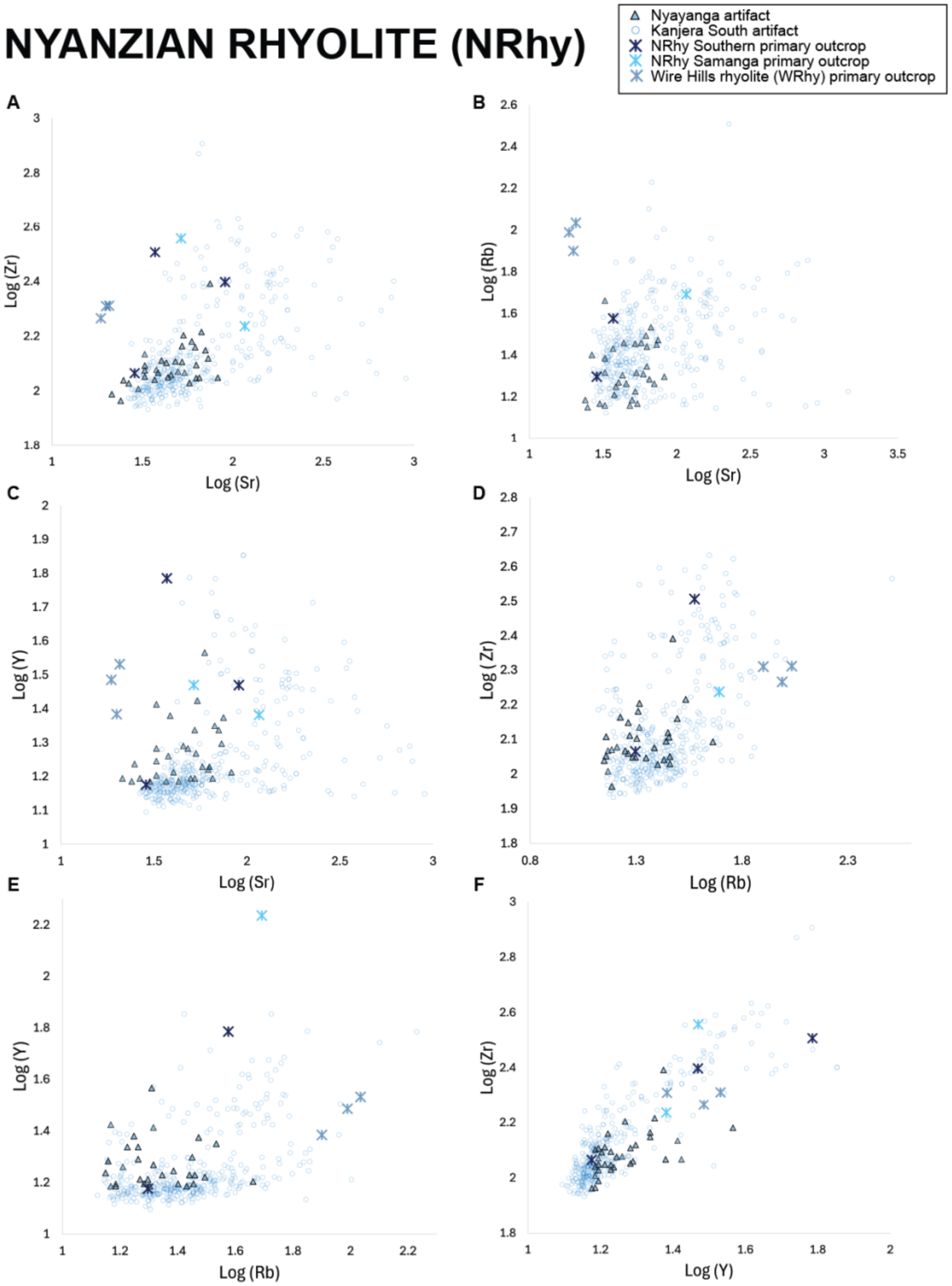

Fig. S6. Bivariate plots of rubidium (Rb), strontium (Sr), yttrium (Y), and zirconium (Zr) logarithmically transformed element concentrations for Nyanzian rhyolite. The geochemistry of Nyanzian rhyolite artifacts (NRhy) from Nyayanga (triangles, n = 34) and Kanjera South (open circles, n = 356) are plotted with samples from Wire Hills (grey), Southern (dark blue), and Samanga (light blue) primary outcrops of Nyanzian rhyolite. A. The ratio of $\log_{10}$ (Sr) (x-axis) and $\log_{10}$ (Zr) (y-axis) concentrations in parts per million (ppm) for all artifacts attributed to quartzite from the Nyayanga (n = 33) and Kanjera South (n = 356) assemblages. B. The ratio of $\log_{10}$ (Sr) (x-axis) and $\log_{10}$ (Rb) (y-axis) concentrations in parts per million (ppm) for all artifacts attributed to quartzite from the Nyayanga (n = 32) and Kanjera South (n = 356) assemblages. C. The ratio of $\log_{10}$ (Sr) (x-axis) and $\log_{10}$ (Y) (y-axis) concentrations in parts per million (ppm) for all artifacts attributed to quartzite from the Nyayanga (n = 33) and Kanjera South (n = 356) assemblages. D. The ratio of $\log_{10}$ (Rb) (x-axis) and $\log_{10}$ (Zr) (y-axis) concentrations in parts per million (ppm) for all artifacts attributed to quartzite from the Nyayanga (n = 32) and Kanjera South (n = 356) assemblages. E. The ratio of $\log_{10}$ (Rb) (x-axis) and $\log_{10}$ (Y) (y-axis) concentrations in parts per million (ppm) for all artifacts attributed to quartzite from the Nyayanga (n = 31) and Kanjera South (n = 356) assemblages. F. The ratio of $\log_{10}$ (Y) (x-axis) and $\log_{10}$ (Zr) (y-axis) concentrations in parts per million (ppm) for all artifacts attributed to quartzite from the Nyayanga (n = 33) and Kanjera South (n = 356) assemblages.

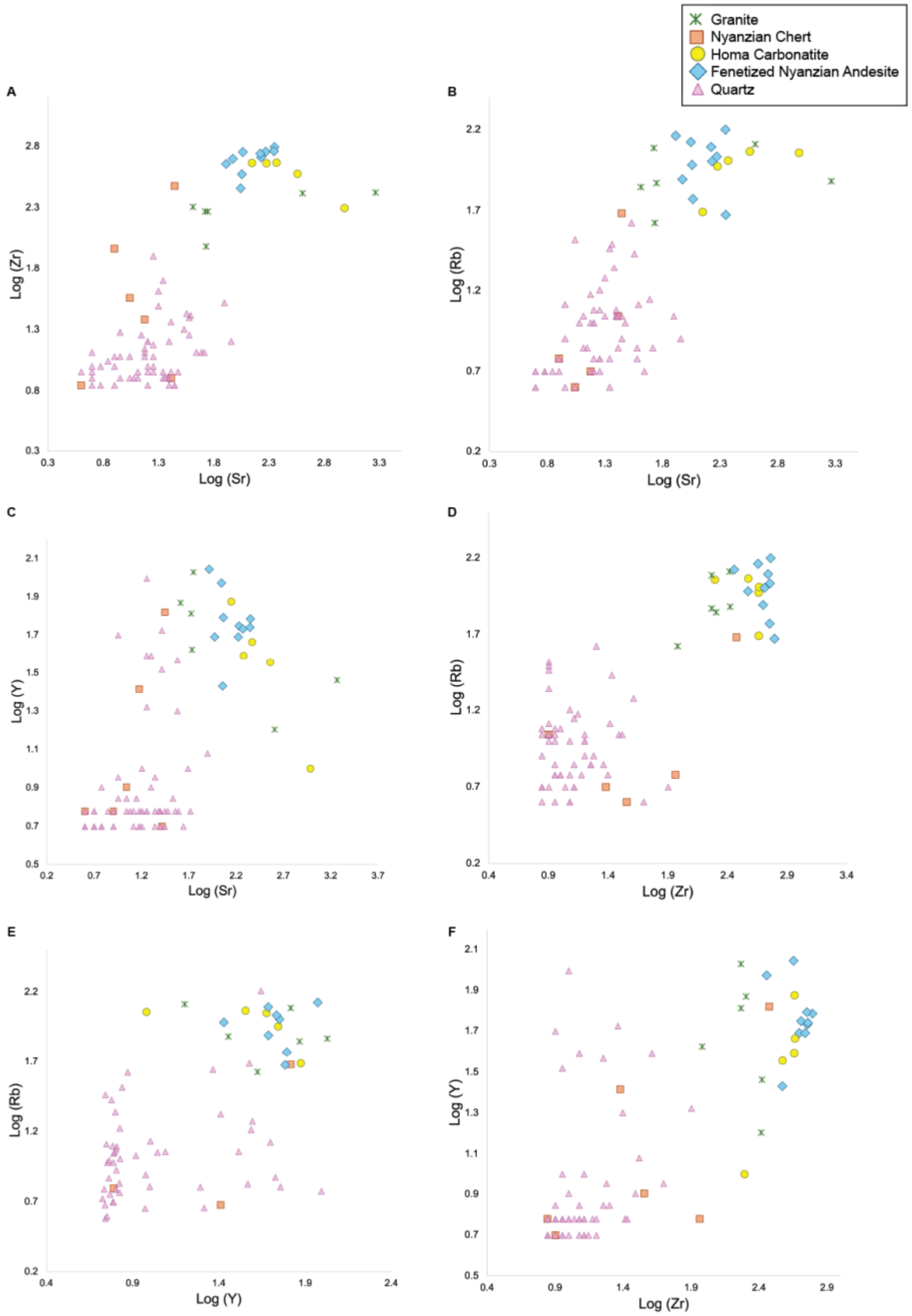

Fig. S7. Bivariate plots of trace element concentrations for other raw materials in the Nyayanga assemblage. All raw material groups with at least four artifacts analyzed using ED-XRF are shown on plots. This included granite (green) quartz (pink), Nyanzian chert (orange), Homa carbonatite (yellow), and fenetized Nyanzian andesite (blue). Raw materials with less than four artifacts run with ED-XRF were omitted (Bukoban felsite, Homa phonolite, Nyanzian andesite, and feldspar porphery). A. The ratio of $\log_{10}$ (Sr) (x-axis) and $\log_{10}$ (Zr) (y-axis) concentrations in parts per million (ppm) for all Nyayanga artifacts attributed to granite ($n = 6$) quartz ($n = 60$), Nyanzian chert ($n = 6$), Homa carbonatite ($n = 5$), and fenetized Nyanzian andesite ($n = 10$). B. The ratio of $\log_{10}$ (Sr) (x-axis) and $\log_{10}$ (Rb) (y-axis) concentrations in parts per million (ppm) for all Nyayanga artifacts attributed to granite ($n = 6$) quartz ($n = 58$), Nyanzian chert ($n = 5$), Homa carbonatite ($n = 5$), and fenetized Nyanzian andesite ($n = 10$). C. The ratio of $\log_{10}$ (Sr) (x-axis) and $\log_{10}$ (Y) (y-axis) concentrations in parts per million (ppm) for all Nyayanga artifacts attributed to granite ($n = 6$) quartz ($n = 63$), Nyanzian chert ($n = 6$), Homa carbonatite ($n = 5$), and fenetized Nyanzian andesite ($n = 10$). D. The ratio of $\log_{10}$ (Zr) (x-axis) and $\log_{10}$ (Rb) (y-axis) concentrations in parts per million (ppm) for all Nyayanga artifacts attributed to granite ($n = 6$) quartz ($n = 56$), Nyanzian chert ($n = 5$), Homa carbonatite ($n = 5$), and fenetized Nyanzian andesite ($n = 10$). E. The ratio of $\log_{10}$ (Y) (x-axis) and $\log_{10}$ (Rb) (y-axis) concentrations in parts per million (ppm) for all Nyayanga artifacts attributed to granite ($n = 6$) quartz ($n = 55$), Nyanzian chert ($n = 5$), Homa carbonatite ($n = 5$), and fenetized Nyanzian andesite ($n = 10$). F. The ratio of $\log_{10}$ (Zr) (x-axis) and $\log_{10}$ (Y) (y-axis) concentrations in parts per million (ppm) for all Nyayanga artifacts attributed to granite ($n = 6$) quartz ($n = 57$), Nyanzian chert ($n = 6$), Homa carbonatite ($n = 5$), and fenetized Nyanzian andesite ($n = 10$).

Table S1. The composition of nonlocal and local rocks in conglomerates surveyed in this study and in Braun et al. 2008 (1). Conglomerates are listed in order from east to west. Rocks are considered local if they derive from primary sources found on the Homa Peninsula. Rocks are considered nonlocal if they derive from primary sources found east of the Peninsula.

Conglomerate	Drainage system	Approximate Age	Local rocks (%)	Nonlocal rocks (%)
Nyayanga	Homa Mountain	Pliocene (Unit NY-1, Rawe Fm)	100	0
Komullo	Homa Mountain	Plio-Pleistocene	99	1
Dago	Homa Mountain	Pliocene (Unit NY-1, Rawe Fm)	98	2
Onyango	Homa Mountain	Unknown	100	0
Homa Lime	Homa Mountain	Pliocene (Homa Fm)	93	7
KCENCON1	Homa Mountain	Pliocene (Rawe Fm)	100	0
KWESTCON3	Homa Mountain	Pleistocene (Apoko Fm)	100	0
KWESTCON1	Homa Mountain	Pliocene (Homa Fm)	100	0
KWESTCON2	Homa Mountain	Pleistocene (Apoko Fm)	99	0
Bala	Homa Mountain	Pliocene (Homa Fm)	97	3
KECON3	Homa Mountain	Pliocene (Abundu Fm)	98	0
KECON2	Homa Mountain	Pleistocene (Apoko Fm)	97	0
KECON4	Homa Mountain	Pleistocene (Kasibos)	98	2
RDRVCON1	Homa Mountain	Pliocene (Rawe? Abundu?)	100	0
RDRVCON2	Homa Mountain	Pleistocene (Apoko Fm)	97	0
Luanda	Homa Mountain	Pliocene (Homa Fm)	94	6
Oboro	Awach	Modern	23	77
AWACON1	Awach	Pleistocene	5	84
AWACON2	Awach	Pleistocene	0	78
NYAPCON2	Nyapetho	Pleistocene	0	79
Sare	Awach	Pleistocene	0	100
AWACON3	Awach	Modern	0	45

Table S2. The relative standard deviation (%RSD) in elements Rubidium (Rb), Strontium (Sr), Ytrium (Y) and Zirconium (Zr) measured across three faces of the same artifact. Artifacts are categorized according to whether they were manufactured from Bukoban quartzite, Nyanzian rhyolite, fenetized Nyanzian rhyolite. All other raw materials are grouped as "Other."

	n	Rb %RSD	Sr %RSD	Y %RSD	Zr %RSD
Bukoban quartzite	86	23.4%	30.2%	28.7%	29.6%
Nyanzian rhyolite	35	24.2%	30.4%	37.3%	33.6%
Fenetized Nyanzian rhyolite	89	20.1%	28.7%	26.4%	29.1%
Other	106	26.2%	29.7%	28.8%	29.6%
Total	316	23.4%	30.2%	28.7%	29.6%

Table S3. The calculated detection limit for elements Rubidium (Rb), Strontium (Sr), Ytrium (Y) and Zirconium (Zr) in parts per million (ppm). The instrument detection limit for each element was calculated based on the background count rate, the concentration, and the peak area of each element.

Element	Detection limit (ppm)	Average detection limit (ppm)	St. dev. detection limit
Rb	4	3.66	1.28
Sr	4	4.12	0.47
Y	5	5.45	0.66
Zr	7	6.99	2.87

Table S4. USGS certified concentrations in parts per million (ppm) of Rubidium (Rb), Strontium (Sr), Ytrium (Y) and Zirconium (Zr) measured for USGS reference standards used for calibration. The USGS reference standards used for calibration were W-2, BHVO-1, SCo-1, BIR-1 and RGM-1.

* = information value

Standard		Rb (ppm)	Sr (ppm)	Y (ppm)	Zr (ppm)
BHVO-1	Basalt, Hawaiian Volcano	11 +/- 2	403 +/- 25	28 +/- 2	179 +/- 21
RGM-1	Rhyolite, Glass Mountain	150 +/- 8	110 +/- 10	25*	220 +/- 20
BIR-1	Icelandic Basalt	-	110 +/- 2	16 +/- 1	18 +/- 1
SCo-1	Cody Shale	110 +/- 4	170 +/- 16	26 +/- 4	160 +/- 30
W-2	Diabase	21 +/- 1.1	190 +/- 3	23 +/- 1.6	100+/- 4

Table S5. Recommended concentrations and measured concentrations of Rubidium (Rb), Strontium (Sr), Ytrium (Y) and Zirconium (Zr) measured for USGS standards BHVO-1 and BIR-1. The standards BHVO-1 and BIR-1 were run as unknowns following calibration with the W-2, SCO-1, and RGM-1 standards to evaluate the accuracy of elemental values in this experimental setup. Measured and recommended values are reported in parts per million (ppm).

Standard		Rb (ppm)	Sr (ppm)	Y (ppm)	Zr (ppm)
BHVO-1	Recommended values	11 +/- 2	403 +/- 25	28 +/- 2	179 +/- 21
	Observed values	4	358	30	204
BIR-1	Recommended values	N/A	110 +/- 2	16 +/- 1	18 +/- 1
	Observed values	4	113	14	17

Captions for excel supplementary data files S1-S4

Excel data file S1: Raw trace element (Rb, Sr, Y, Zr) geochemistry reported in parts per million for artifacts in the Nyayanga assemblage included in analysis. Any value below the detection limit is reported as "BDL" (below detection limit).

Excel data file S2: Technological variables for flakes in the Nyayanga assemblage.

Excel data file S3: Logarithmically transformed trace element (Rb, Sr, Y, Zr) values for Nyayanga artifacts, Kanjera North artifacts, and raw material sources reported in this study. The elemental compositions determined for Nyayanga artifacts and clasts from conglomerates analyzed in this study were calibrated and transformed according to Finestone et al. (53) to allow comparability with Kanjera North artifacts and sources previously analyzed by Braun et al. (1). Any missing values indicate that the element concentration was below the detection limit.

Excel data file S4: Technological variables for cores in the Nyayanga assemblage.
